# Altered nucleosome positions in maize haplotypes and mutants of a subset of SWI/SNF‐like proteins

**DOI:** 10.1002/pld3.19

**Published:** 2017-10-16

**Authors:** Linda K. Stroud, Karen M. McGinnis

**Affiliations:** ^1^ Department of Biological Science Florida State University Tallahassee FL USA

**Keywords:** chromatin, chromatin remodelers, nucleosome position, transcription start site, *Zea mays*

## Abstract

Chromatin remodelers alter DNA–histone interactions in eukaryotic organisms and have been well characterized in yeast and Arabidopsis. While there are maize proteins with similar domains as known remodelers, the ability of the maize proteins to alter nucleosome position has not been reported. Mutant alleles of several maize proteins (RMR1, CHR101, CHR106, CHR127, and CHR156) with similar functional domains to known chromatin remodelers were identified. Altered gene expression of *Chr101*,* Chr106*,* Chr127*, and *Chr156* was demonstrated in plants homozygous for the mutant alleles. These mutant genotypes were subjected to nucleosome position analysis to determine whether misregulation of putative maize chromatin proteins would lead to altered DNA–histone interactions. Nucleosome position changes were observed in plants homozygous for *chr101*,* chr106*,* chr127*, and *chr156* mutant alleles, suggesting that CHR101, CHR106, CHR127, and CHR156 may affect chromatin structure. The role of RNA polymerases in altering DNA–histone interactions was also tested. Changes in nucleosome position were demonstrated in homozygous *mop2‐1* individuals. These changes were demonstrated at the *b1 tandem repeats* and at newly identified loci. Additionally, differential DNA–histone interactions and altered gene expression of putative chromatin remodelers were demonstrated between different maize haplotypes.

## 1 INTRODUCTION

Eukaryotic DNA is organized into a compact arrangement of DNA and proteins referred to as chromatin. The basic unit of chromatin is the nucleosome, which is made up of 150 base pairs of DNA wrapped 1.65 times around a histone octamer that is made up of two each H2A, H2B, H3, and H4 histones. DNA is further packaged with the addition of histone H1 and scaffold proteins to linker DNA between nucleosomes. While these DNA–histone interactions are in part determined by the DNA sequence itself, they can also be determined by chromatin remodeling complexes (reviewed by Jiang & Pugh, 2009) that utilize ATP hydrolysis to generate energy for changing DNA–histone interactions (reviewed by Kwon & Wagner, 2007).

Chromatin remodeling is the ATP‐dependent noncovalent alteration of DNA–histone interactions (reviewed by Kwon & Wagner, 2007). Chromatin remodeling is considered biologically significant because it is associated with changes in transcriptional activity and other phenotypes (reviewed by Kwon & Wagner, 2007). One of the four major families of chromatin remodeling complexes is switching defective/sucrose nonfermenting (SWI/SNF) (reviewed by Clapier & Cairns, 2009). The motor proteins of SWI/SNF belong to the SNF2 family of proteins and are characterized by their conserved SNF2_N and Helicase_C domains (Eisen, Sweder, & Hanawalt, 1995; Flaus, Martin, Barton, & Owen‐Hughes, 2006). Several of the SNF2 motor proteins have demonstrated chromatin remodeling ability, including *S. cerevisiae* (Sc) ScSNF2 (Laurent, Treich, & Carlson, 1993), ScRAD54 (Alexeev, Mazin, & Kowalczykowski, 2003; Petukhova, Van Komen, Vergano, Klein, & Sung, 1999), and *A. thaliana* (At) AtDDM1 (Brzeski & Jerzmanowski, 2003). AtDDM1 has also been shown to function in the maintenance of DNA methylation, in genomewide transposon and repetitive sequence methylation, and in the spread of silencing‐related histone modifications (Ito et al., 2015; Jeddeloh, Bender, & Richards, 1998; Jeddeloh, Stokes, & Richards, 1999).

Maize (*Zea mays*) proteins highly similar to the Arabidopsis AtDDM1 (Jeddeloh et al., 1998), AtDRD1 (Kanno et al., 2004), and AtCLSY1 (Smith et al., 2007) have been identified. These include the homologs CHR101 and CHR106 (Li et al., 2014), CHR127 and CHR156, and RMR1 (Hale, Stonaker, Gross, & Hollick, 2007) and CHR167 (Haag et al., 2014). While the biochemical activity of these proteins has not been reported, various phenotypes associated with mutant alleles of some proteins have been described. Among these, the extensively studied RMR1 is highly similar to AtCLSY1 and has been implicated in the spontaneous epigenetic silencing of a transgene (McGinnis, Springer, Lin, Carey, & Chandler, 2006) and the regulation of *b1* and *pl1* transcript abundance (Hale et al., 2007). It has also been found to interact with RNA polymerase IV (PolIV), along with CHR167 (Haag et al., 2014) and AtCLSY1 (Zhang et al., 2013). The maize proteins CHR101 and CHR106 are highly similar to AtDDM1 (Li et al., 2014). Similar to phenotypes described for Arabidopsis *ddm1* homozygous plants (Jeddeloh et al., 1998, 1999), maize mutants defective for CHR106 exhibit reduced CHH and CHG (H = A, C, or T) methylation (Li et al., 2014). The maize proteins CHR127 and CHR156 are highly similar to AtDRD1, but mutant alleles have not been uncovered in published forward genetic screens based on disrupted epigenetic silencing in maize (Dorweiler et al., 2000; Hollick & Chandler, 2001; Hollick, Kermicle, & Parkinson, 2005; Madzima, Mills, Gardiner, & McGinnis, 2011; Sidorenko et al., 2009; Stonaker, Lim, Erhard, & Hollick, 2009). Similar to its Arabidopsis homolog (Law et al., 2010), CHR127 interacts with RNA polymerase V (PolV) (Haag et al., 2014). These phenotypic similarities suggest that these maize proteins may have similar functions to their Arabidopsis homologs.

While chromatin remodelers have been extensively studied in several organisms, the activities of such proteins have not been investigated in maize yet. Therefore, this study investigated nucleosome positioning and its relationship to transcriptional regulation by focusing on the transcription start site (TSS) regions of a random selection of 400 classical genes on a microarray. Although sequencing has become a favored approach with the increasing availability of high‐throughput sequencing, this microarray provided a cost‐effective way to identify changes at several loci in uncharacterized genotypes. Herein, several mutant maize alleles of putative chromatin remodelers were identified and tested with nucleosome protection assay to identify nucleosome position changes.

## 2 MATERIALS AND METHODS

### 2.1 Phylogeny

The Saccharomyces Genome Database (SGD, http://www.yeastgenome.org/) was used to acquire the amino acid sequences and identifiers (ID) for ScSNF2 (SGD ID S000005816) and ScRAD54 (SGD ID S000003131). The Arabidopsis amino acid sequences were acquired from The Arabidopsis Information Resource (TAIR, https://www.arabidopsis.org/), along with the Arabidopsis Genome Initiative (AGI) identifiers for AtDDM1 (AGI ID At5g66750), AtCLSY1 (AGI ID At3g42670), and AtDRD1 (AGI ID At2g16390). These proteins were queried against *Zea mays* using NCBI's BLASTP (Altschul, Gish, Miller, Myers, & Lipman, 1990) to identify the maize proteins within the top five percent identity for each query. The Pfam SNF2_N and Helicase_C domains of the yeast, Arabidopsis, and selected maize proteins were identified by BLASTP and MUSCLE Aligned using the software Geneious (http://www.geneious.com/). The percent identities at each amino acid were downloaded and averaged to determine domain conservation.

Phylogenetic tree was generated, and the number of amino acid substitutions was determined in the Helicase_C domains using MEGA7 (Kumar, Stecher, & Tamura, 2016). The evolutionary history was inferred by using the maximum‐likelihood method based on the JTT matrix‐based model (Jones, Taylor, & Thornton, 1992). The Archaea *Haloarcula californiae* ATP‐dependent helicase (NCBI accession WP_007188160) was used as the outgroup.

### 2.2 Plant materials

Seeds for the wild types B73 and W22 were acquired from the Maize Genetics Cooperation Stock Center (http://maizecoop.cropsci.uiuc.edu/) and have been maintained through self and sibling pollination. Seeds for *chr101‐m1* (B73 background), *chr101‐m3* (B73 background), *chr106‐m1* (B73 background), and *chr106‐T11* (W22 background) were generously donated by the Springer Lab (University of Minnesota) and were originally described by Li et al. (2014). The 5′UTR transposon insertion alleles *chr127‐m1* (mu1048254) and *chr156‐m1* (mu1055648) (McCarty & Meeley, 2009; Settles et al., 2007) were identified through MaizeGDB (http://www.maizegdb.org/) and were acquired from the Maize Genetics Cooperation Stock Center. The *chr127‐m1* and *chr156‐m1* genotypes are in the W22 background and have been maintained through self or sibling pollinations. For *rmr1‐1* (mixed background), seed stocks from segregating families of homozygous mutants and heterozygotes were used (Hollick, Patterson, Asmundsson, & Chandler, 2000). For *mop2‐1* (W23/K55 background), homozygous mutants and wild types from segregating families were used (Sidorenko et al., 2009). For each plant, seedling leaf tips were collected for genotyping purposes. The eighth leaf of each plant was also collected when the plants were at their V9 stage of development to maintain consistency due to the effects of plant development and differentiation. These leaves were immediately flash frozen in liquid nitrogen and stored at −80°C until nuclei or RNA extraction.

### 2.3 DNA extractions and genotyping

Genomic DNA was isolated from approximately 2‐cm^2^ leaf tips of seedlings using 96‐well plate CTAB protocol (Labonne, Dorweiler, & McGinnis, 2013). The genotyping primers and detection methods are described for each genotype in Table S2. Sanger sequencing was performed at the Molecular Core Facility (Department of Biological Sciences, Florida State University).

### 2.4 RNA extraction and qRT‐PCR

Plant tissue was ground in liquid nitrogen, and then, RNA was extracted using TRI Reagent (Molecular Research Center, Inc.) according to the manufacturer's instructions. The resulting RNA was DNaseI (Promega) treated at 37°C for 30 min and then cleaned using RNA clean and concentrator‐25 (Zymo Research). Superscript III (Invitrogen) was used to reverse transcribe 1 ug of RNA into cDNA according to the manufacturer's instructions. cDNA was used as a template for qRT‐PCR using gene‐specific primers and *Ubiquitin‐conjugating enzyme* (Sekhon et al., 2011) or 45S rRNA (Hale, Erhard, Lisch, & Hollick, 2009) primers for internal control (Table S3) with SYBR Green Master Mix (Applied Biosystems) per the manufacturer's instructions on a 7500 Fast Real‐Time PCR System (Applied Biosystems). Relative expression changes were calculated using 2^−∆∆Ct^ (Livak & Schmittgen, 2001). All data represent two technical replicates.

### 2.5 Nuclei isolation

Nuclei were isolated from 10 g of leaf tissue as described previously (Vera et al., 2014) with several modifications. Frozen, ground tissue was fixed in ice‐cold modified Apel buffer (20 mm Tris‐HCl pH 7.8, 250 mm sucrose, 5 mm MgCl_2_, 5 mm KCl) and 1% formaldehyde by gently stirring for 10 min on ice. Fixation was stopped by stirring with 125 mm glycine for 5 min. The cells were lysed by continuing to stir on ice for 5 min with 0.25% Triton X‐100 (EMD Millipore) and 0.1% BME. This mixture was filtered through two layers of Miracloth (Calbiochem) and suspended over 50% Percoll density gradient media (GE Healthcare) in Apel buffer. The nuclei were floated by centrifugation at 2000 *g* for 15 min at 4°C in a swinging‐bucket centrifuge. The band of nuclei floating on top of the Percoll were transferred to new ice‐cold tubes and resuspended in a minimum of twice the nuclei‐band volume of MNase digestion buffer (50 mm Tris‐HCl pH 7.5, 320 mm sucrose, 4 mm MgCl_2_, and 1 mm CaCl_2_). Nuclei were pelleted by centrifugation at 500 *g* for 15 min, resuspended in 1 ml of MNase digestion buffer, aliquoted and flash frozen in liquid nitrogen, and stored at −80°C until use.

### 2.6 Nuclei quantification, MNase digestion, and nucleosomal DNA isolation

Quantification of nuclei, MNase digestion, and mono‐/dinucleosomal DNA purification were performed as described previously (Labonne et al., 2013).

### 2.7 Analysis of nucleosomal DNA by microarray

Digested nucleosomal DNA and reference undigested DNA were labeled with Cy3 and Cy5 (NimbleGen), respectively, according to the manufacturer's instructions. The labeled DNA were then hybridized to a ~400 classical gene transcription start site microarray (Labonne et al., 2013) and probed according to the manufacturer's instructions (NimbleGen) in the Molecular Core Facility (Department of Biological Sciences, Florida State University). DEVA software (NimbleGen) generated normalized ratio GFF files that were analyzed using R (R Development Core Team) and the DrawGff package (http://fincher.github.io/DrawGff/).

Initially, to eliminate highly polymorphic regions, the raw GFF files for the genomic DNA of B73 and W22 were compared. 364 of the 3000‐bp probe regions were identified with no significant difference (Student's *t* test, *p* > .05) for at least 50% of their probes. Only these 364 regions were included in subsequent analysis. To determine the statistical significance of the identified changes in nucleosome position, Student's t test statistical analysis was performed at each probe using the raw GFF files for the nucleosomal and reference data. Regions having a significant difference (Student's *t* test, *p* ≤ .05) between wild‐type and mutant nucleosomal DNA while having no significant difference (Student's *t* test, *p* > .05) between the wild‐type and mutant reference sequences through a minimum of 140‐bp continuous probe coverage were identified.

### 2.8 Analysis of nucleosomal DNA by qPCR

Five of the loci (*Stc1_NP1*,* Tdy1_NP1*,* Bx1_NP1*,* Tub2_NP1*, and *Hm1_NP1*) with robust nucleosome position differences between the genotypes identified by microarray analysis were also tested by alternate MNase‐qPCR method (Fig. S4a,b). Two ng of MNase‐digested nucleosomal and reference undigested genomic DNA, prepared as described above, was analyzed using qPCR. Primers spanning the region of significant nucleosome position change were designed for *Fdx3_NP1*,* Stc1_NP1*,* Tdy1_NP1*,* Hm1_NP1*,* Bx1_NP1*, and *Tub2_NP1* (Table S4). A locus (*Mwp1*‐GRMZM2G082264) with a consistent nucleosome position region between genotypes was identified from the microarray analysis, and it was also tested by MNase‐qPCR (Fig. S5) as an internal control. The relative nucleosome position changes for the other loci were calculated using 2^−∆Ct^ (=2^−[Ct(MNase)−Ct(gDNA)]^) and normalizing each 2^−∆Ct^ to that of *Mwp1* (Han et al., 2012). All data represent two technical replicates.

### 2.9 Motif search

The fasta‐get‐markov program was acquired through the MEME Suite (http://meme-suite.org/) and was used to generate the appropriate Markov model from the transcription start site regions of the ~400 classical genes of the microarray used in this study. This Markov model was used as the background when searching for shared sequence motifs at the altered nucleosome position loci. Regions of the DNA sequences with significant nucleosome position change, as determined by the microarray analysis, were searched for the presence of common motifs using MEME (Bailey & Elkan, 1994) of the MEME Suite. Motif‐1 was further analyzed by submitting it to the TOMTOM (Gupta, Stamatoyannopolous, Bailey, & Noble, 2007) application for comparison to known maize and Arabidopsis transcription factor binding motifs (O'Malley et al., 2016; Yu et al., 2015) using a significance of *E*‐value ≤0.05. Motif‐1 was also scanned at the *b1 tandem repeats*, accession number AY078063 (Stam, Belele, Ramakrishna et al., 2002) using FIMO (Grant, Bailey, & Noble, 2011) of the MEME Suite.

## 3 RESULTS

### 3.1 Identification of putative maize chromatin remodelers

The SNF2 family proteins share conserved SNF2_N and Helicase_C domains (Eisen et al., 1995) and have demonstrated abilities to alter nucleosome position (NP) in yeast (Alexeev et al., 2003; Laurent et al., 1993; Petukhova et al., 1999) and in Arabidopsis (Brzeski & Jerzmanowski, 2003). An alignment analysis (Altschul et al., 1990) was used to identify putative maize chromatin remodelers by querying the database with SNF2 family proteins from *S. cerevisiae* and *A. thaliana*. Maize proteins with E‐values below 0.01 were identified and those with the highest percent identities were selected for further analysis (Table S1). These proteins included the unnamed GRMZM2G083138, GRMZM2G102625, and GRMZM2G108166 proteins and six previously characterized proteins: CHR101, CHR106, CHR127, CHR156, RMR1, and CHR167. Genotypes mutant for CHR101 and its homolog CHR106 have published DNA methylation phenotypes (Li et al., 2014). RMR1, CHR167 and CHR127 interact with RNA polymerases IV or V (Haag et al., 2014) and have another homolog CHR156 (Hale et al., 2007). On average, the identified maize proteins were 32.0% identical to the known yeast and Arabidopsis proteins. As expected for two plant species, Arabidopsis and maize were typically more identical, at an average of 33.4%, than maize and yeast with an average identity of 29.9%.

**Figure 1:**
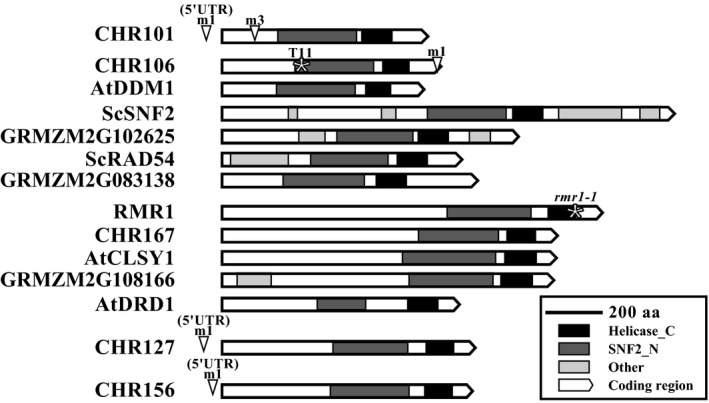
Putative chromatin remodelers and their domains. Scaled diagram of the *Zea mays*,* S. cerevisiae* (Sc), and *A. thaliana* (At) proteins. The white arrows indicate the protein‐coding regions. Shaded blocks indicate Pfam‐identified SNF2_N (dark gray), Helicase_C (black), and additional non‐conserved domains (light gray). Transposon insertions (▽) and point mutations (*) are indicated with the allele names. A scale of 200 amino acids (aa) is indicated

Based upon similarity to the Pfam domains (Finn et al., 2016), SNF2_N and Helicase_C domains were identified for the maize, Arabidopsis, and yeast chromatin proteins (Figure 1). The conservation of these domains among the maize, Arabidopsis, and yeast proteins was investigated through their MUSCLE alignments (Edgar, 2004). The pairwise identities indicated that the Helicase_C domains are more conserved, with an average 37% identity, than the SNF2_N domains, with an average 24% identity (Fig. S1a,b).

Because of strong conservation, the Helicase_C domain was a good candidate for phylogenetic analysis using MEGA7's (Tamura, Stecher, Peterson, Filipski, & Kumar, 2013) maximum‐likelihood (JTT model) method. The evolutionary divergence of the maize and Arabidopsis protein was determined based on the number of amino acid substitutions per site (aas). The maize, Arabidopsis, and yeast proteins were divided into two subgroups. Subgroup 1 included AtDDM1 and the maize homologs CHR101 and CHR106, which diverged 0.10 aas from each other and an average of 0.21 aas from AtDDM1 (Table 1). This group also included GRMZM2G102625, GRMZM2G083138, ScSNF2, and ScRAD54 (Fig. S2). Subgroup 2 included RMR1, CHR167, CHR127, CHR156, GRMZM2G108166, AtDRD1, and AtCLSY1 (Fig. S2). Phylogenetic analysis determined a 0.10 aas divergence between CHR127 and CHR156 and an average divergence of 0.39 aas of these proteins from AtDRD1 (Table 1). CHR101, CHR106, CHR127, CHR156, RMR1, CHR167, GRMZM2G102625, GRMZM2G083138, and GRMZM2G108166 were identified as potential chromatin remodeling proteins and were selected for further analysis.

**Table 1:** Amino acid substitutions per site in the Helicase_C domain

Helicase_C	AtDDM1	RMR1	CHR101	CHR106	CHR127	CHR156	ScRAD54	ScSNF2	GRMZM 2G083138	GRMZM 2G102625	AtDRD1	AtCLSY1	GRMZM 2G108166	CHR167	WP_007188160
AtDDM1	0.00														
RMR1	1.34	0.00													
CHR101	0.19	1.26	0.00												
CHR106	0.22	1.25	0.10	0.00											
CHR127	1.87	0.87	1.73	1.71	0.00										
CHR156	1.82	0.84	1.65	1.64	0.10	0.00									
ScRAD54	1.13	1.42	1.07	1.09	1.65	1.57	0.00								
ScSNF2	0.73	1.54	0.73	0.73	2.06	1.89	1.25	0.00							
GRMZM2G083138	1.24	1.57	1.29	1.27	1.67	1.64	0.33	1.27	0.00						
GRMZM2G102625	0.55	1.55	0.53	0.50	1.79	1.73	1.23	0.40	1.14	0.00					
AtDRD1	1.83	0.91	1.70	1.75	0.37	0.40	1.66	1.91	1.75	1.78	0.00				
AtCLSY1	1.41	0.97	1.38	1.44	0.99	0.95	1.34	1.67	1.57	1.61	1.01	0.00			
GRMZM2G108166	1.60	0.99	1.55	1.61	0.91	0.89	1.41	1.64	1.61	1.75	0.89	0.47	0.00		
CHR167	1.30	0.47	1.36	1.39	0.79	0.85	1.34	1.53	1.46	1.56	0.91	1.12	0.96	0.00	
WP_007188160	2.00	1.90	1.92	1.97	2.02	1.99	2.09	1.98	1.87	1.92	2.00	1.93	1.96	1.82	0.00

The number of amino acid substitutions per site between sequences of the Helicase_C domains of each maize, *A. thaliana* (At), and *S. cerevisiae* (Sc) proteins are shown. Duplicate values in table were left blank.

### 3.2 Identification of alleles with altered gene expression of maize putative chromatin proteins

**Figure 2:**
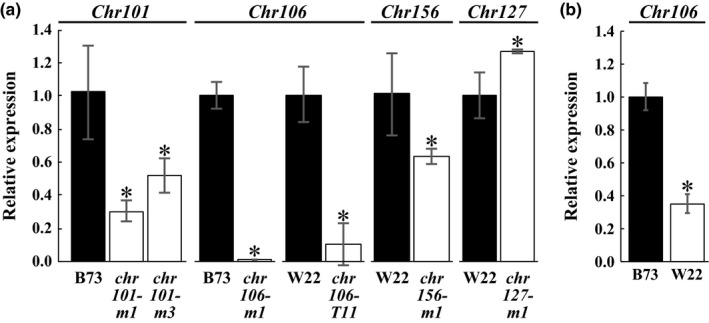
Gene expression of putative chromatin protein‐encoding genes in different genotypes. (a) Relative expression levels of the different chromatin proteins (above bars) were determined by qRT‐PCR and calculated as 2^−∆∆Ct^. Gene expression was determined in wild type (black bar) and homozygous mutant (white bar) individuals, and expression level of the non‐mutant gene in the appropriate wild genotype was set to 1 for each comparison. The data represent averages ±*SD* (error bars) of three replicates that were normalized to *Ubiquitin‐conjugating enzyme*. Student's t test (*p* ≤ .05) was used to identify statistically significant changes between mutant and WT (*). (b) Relative expression levels of *Chr106* in B73 (black bar) and W22 (white bar) plants were determined by qRT‐PCR and calculated as 2^−∆∆Ct^. The data represent averages ±*SD* (error bars) of three pools of three biological replicates that were normalized to *Ubiquitin‐conjugating enzyme*. Student's t test (*p* ≤ .05) was used to identify statistically significant change between the haplotypes (*)

To further investigate the function of the putative maize chromatin proteins, alleles of *Chr101*,* Chr106*,* Chr127*,* Chr156*, and *Rmr1* were identified in reverse and forward genetics resources (Hollick & Chandler, 2001; McCarty & Meeley, 2009; Settles et al., 2007; Till et al., 2004). At the time of the search, alleles could not be identified for *Chr167*, GRMZM2G102625, GRMZM2G083138, and GRMZM2G108166. However, mutant alleles for both subgroups of the phylogenetic tree were identified. Mutant alleles of *chr101* and *chr106* include transposon insertions in the 5′untranslated region (UTR, *chr101‐m1*) and exons (*chr101‐m3, chr106‐m1*), as well as a nonsense point mutation (*chr106‐T11*, Figure 1). Plants homozygous for these alleles were already demonstrated to have altered DNA methylation, and *chr101 chr106* double mutants exhibited lethality (Li et al., 2014). The gene expression level of *Chr101* and *Chr106* had not been reported for these genotypes. Based upon real‐time quantitative reverse transcription‐PCR (qRT‐PCR) analysis, gene expression was significantly reduced in all four homozygous mutants compared to wild type (Figure 2a). Gene expression of *Chr101* was reduced to approximately half of the wild type in both the *chr101‐m1* and *chr101‐m3* homozygous mutants. The homozygous mutants *chr106‐m1* and *chr106‐T11* appeared to be null alleles with the gene expression of *Chr106* being reduced to nearly zero (Figure 2a).

For *Chr127* and *Chr156*, 5′UTR transposon insertion alleles (*chr127‐m1, chr156‐m1*) were identified (Figure 1). These alleles did not have any observable vegetative or reproductive phenotypes (data not shown), possibly due to *chr127‐m1* and *chr156‐m1* being mutations with weak expressivity or because of genetic redundancy. Based on qRT‐PCR analysis, the *chr156‐m1* homozygous mutant exhibited an approximately one third reduction from wild‐type gene expression (Figure 2a), and the *chr127‐m1* homozygous mutants exhibited approximately one third increased gene expression compared to wild type. Elevated gene expression due to transposon insertion has been demonstrated to lead to phenotypic abnormalities (Barkan & Martienssen, 1991; Robbins, Sekhon, Meeley, & Chopra, 2008), indicating that this allele might be useful for studying CHR127 function.

For RMR1, the well‐characterized *rmr1‐1* mutant allele was chosen. It includes a point mutation in its Helicase_C domain (Figure 1) and was previously characterized as a loss‐of‐function allele with detectable phenotypes (Hale et al., 2007). While this allele did not show any significant changes in gene expression (data not shown), the molecular lesion presumably alters protein structure and function.

### 3.3 The inbred lines B73 and W22 exhibit differential nucleosome positions

The mutant alleles of *chr106*,* chr106‐m1* and *chr106‐T11*, were isolated from mutant stocks of different genetic backgrounds, B73 and W22 (Li et al., 2014). Genetic diversity has been demonstrated between the maize inbred Stiff Stalk haplotypes, including B73, and Non‐Stiff Stalk haplotypes, including W22 (Liu et al., 2003). Nucleosome positions of B73 and W22 were compared to each other to identify haplotype‐specific differences. The nucleosome position comparison was performed using microarray analysis of nucleosome‐sized DNA relative to undigested DNA in a 3000‐bp window of the transcription start site (TSS) region of 400 classical genes (Labonne et al., 2013).

As this analysis is highly sensitive to sequence polymorphisms, and the different haplotypes are known to be polymorphic (van Heerwaarden, Hufford, & Ross‐Ibarra, 2012), regions with substantial genetic variation between the relevant genotypes were excluded from this and all subsequent analysis, leaving 364 loci that are amenable to study with this technique. To detect nucleosome position changes, the log2 ratio of the signal intensities of the different genotypes was plotted using an R script (Druliner et al., 2013) and was observed for changes between haplotypes. The hybridization signal pattern for each genotype was compared against the hybridization signal of the same genotype genomic DNA to identify “peaks” corresponding to strongly positioned nucleosomes and “valleys” indicating a lack of positioned nucleosomes in that genomic position. In comparing nucleosome positions between the two haplotypes, nucleosome positions were consistent at most genomic locations, with a microarray‐wide Pearson correlation of 0.85. However, there were some discrete changes at specific loci, suggesting localized changes in chromatin structure.

**Figure 3:**
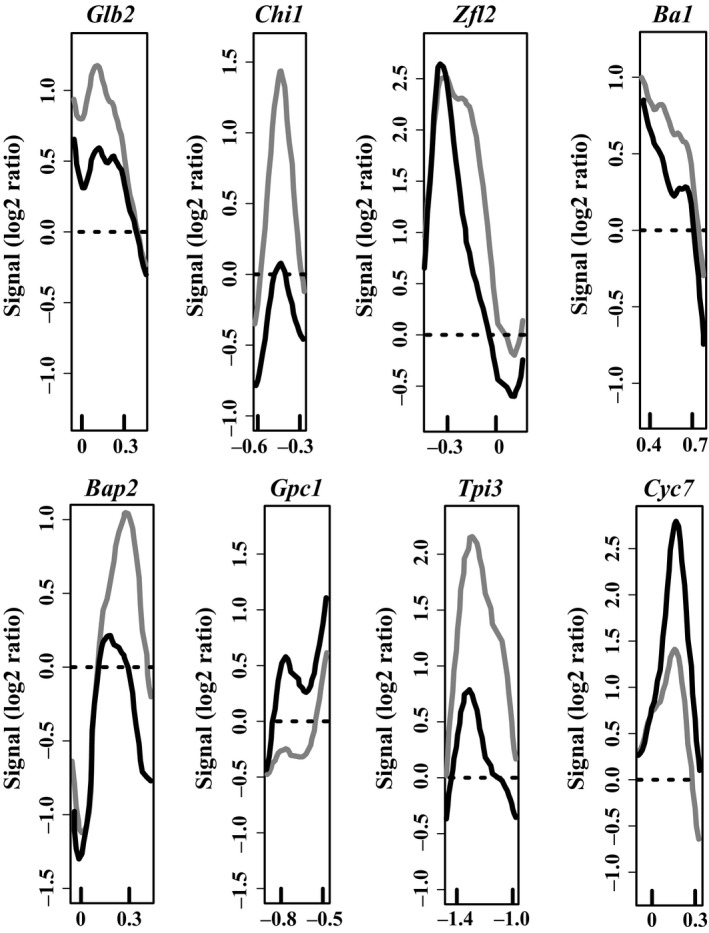
Nucleosome position differences between the B73 and W22 haplotypes. Comparison of the nucleosome position in the haplotypes B73 (gray line) and W22 (black line). Genes are all oriented according to a left‐to‐right transcriptional direction, with the nucleotide position in kb relative to the transcription start site (0) indicated on the x‐axes. Relative fluorescent signals are indicated on the y‐axes (Signal (log2 ratio)). Three pools of three biological replicates were averaged for each plot

Between B73 and W22, eight loci with statistically significant nucleosome position changes longer than 140 bp were detected (Figure 3; Table 2). A positioned nucleosome was gained in W22 at two loci (*Gpc1_NP1* and *Cyc7_NP1*) and in B73 at the other six loci. As differential expression of some genes has been demonstrated between the two haplotypes (Dong et al., 2016), gene expression of *Chr106* was analyzed in B73 and W22. The qRT‐PCR comparison of the two haplotypes demonstrated the significantly reduced gene expression of *Chr106* in W22 compared to B73 (Figure 2b), suggesting that there is differential chromatin protein activity between haplotypes of maize.

**Table 2:** Maize loci with detectable nucleosome position differences between B73 and W22 haplotypes

Gene name	Gene ID	NP change name	Haplotype with NP gain	Motif‐1
*Glb2*	GRMZM2G026703	*Glb2_NP1*	B73	
*Chi1*	GRMZM2G155329	*Chi1_NP1*	B73	
*Zfl2*	GRMZM2G180190	*Zfl2_NP1*	B73	
*Ba1*	GRMZM2G397518	*Ba1_NP1*	B73	
*Bap2*	GRMZM2G152655	*Bap2_NP1*	B73	
*Gpc1*	GRMZM2G046804	*Gpc1_NP1*	W22	1
*Tpi3*	GRMZM2G018177	*Tpi3_NP1*	B73	
*Cyc7*	GRMZM2G310115	*Cyc7_NP1*	W22	

The names and IDs of maize genes with demonstrated nucleosome position (NP) change between the B73 and W22 haplotypes are listed. The haplotype with the gained nucleosome position (haplotype with NP gain) is indicated as well as the number of Motif‐1 occurrences.

### 3.4 Nucleosome position is altered in plants homozygous for mutant alleles of putative chromatin proteins

One possible phenotype associated with disrupted chromatin remodeler activity is a change in nucleosome position in transcriptional regulatory regions of the genome (reviewed by Jiang & Pugh, 2009). To identify potential chromatin structure phenotypes, wild‐type plants and homozygous mutants for *rmr1*,* chr101*,* chr106*,* chr127,* and *chr156* were compared using microarray analysis as described for the B73 and W22 haplotypes. Nucleosome positions were consistent between genotypes at most genomic locations, with a microarray‐wide Pearson correlation average of 0.93 between mutant and wild‐type genotypes. However, there were some discrete changes at specific loci, suggesting that loss of the respective protein activity may induce localized, rather than genomewide changes in chromatin structure. Nucleosome position patterns were compared for each mutant genotype against its corresponding wild‐type nucleosome position map and statistically significant nucleosome position changes longer than 140 bp were detected. Among all the genotypes tested, a total of 23 loci with altered nucleosome positions were identified (Table 3). Because a genotype‐ or lineage‐specific transposon or other sequence polymorphism could lead to some of the detected differences, a control of nucleosomal DNA by genomic DNA was used to minimize false positives. Additionally, the use of multiple alleles with distinct genetic pedigrees is also expected to reduce the likelihood of this, suggesting that many or most of these differences in nucleosome position are true, genotype‐specific changes. These loci did not seem to belong to any one consistent gene ontology (GO) group (data not shown), suggesting that the effects were not limited to any specific categories of biological function.

**Table 3:** Maize loci with detectable nucleosome position differences in different genotypes

Gene name	Gene ID	NP change name	Genotypes with altered NP	Genetic background	NP in mutants	% mCHH^a^	CHHi location^a^	More correlated genotype to NOL	Motif‐1
*Fdx3*	GRMZM2G053458	*Fdx3_NP1*	chr101‐m1 chr101‐m3 chr106‐T11	B73 B73 W22	Gain Gain Gain	NA^b^	NA^b^	M M M	2 2 1
*Adf1*	GRMZM2G117603	*Adf1_NP1*	chr101‐m1 chr101‐m3	B73 B73	Loss Loss	66.7	5′ end	M M	
*Pdc2*	GRMZM2G038821	*Pdc2_NP1*	chr101‐m1 chr101‐m3	B73 B73	Loss Loss	32.3	3′ end	M M	
*Pdc2*	GRMZM2G038821	*Pdc2_NP2*	chr101‐m1 chr101‐m3	B73 B73	Gain Gain	32.3	3′ end	M M	
*Stc1*	GRMZM2G177098	*Stc1_NP1*	chr106‐T11 chr127‐m1	W22 W22	Loss Loss	25.0	3′ end	M M	
*Tpi3*	GRMZM2G018177	*Tpi3_NP1*	*chr106‐T11*	W22	Gain	NA^b^	NA^b^	W	
*Tdy1*	GRMZM2G321778	*Tdy1_NP1*	*chr127‐m1*	W22	Gain	33.3 77.7	3′ end 5′ end	M	2
*Bx1*	GRMZM2G085381	*Bx1_NP1*	*chr156‐m1*	W22	Loss	NA^b^	NA^b^	M	
*Bx1*	GRMZM2G085381	*Bx1_NP2*	*chr156‐m1*	W22	Loss	NA^b^	NA^b^	M	1
*Tub2*	GRMZM2G334899	*Tub2_NP1*	*chr156‐m1*	W22	Loss	NA^b^	NA^b^	M	
*Hm1*	GRMZM5G881887	*Hm1_NP1*	*chr156‐m1*	W22	Loss	77.8	3′ end	W	1
*Pac1*	GRMZM2G058292	*Pac1_NP1*	*chr156‐m1*	W22	Loss	NA^b^	NA^b^	M	3
*Adh2*	GRMZM2G098346	*Adh2_NP1*	*chr156‐m1*	W22	Gain	52.9	3′ end	W	1
*Ms26*	GRMZM2G091822	*Ms26_NP1*	*chr156‐m1*	W22	Loss	NA^b^	NA^b^	M	
*Ms26*	GRMZM2G091822	*Ms26_NP2*	*chr156‐m1*	W22	Loss	NA^b^	NA^b^	M	
*Ts1*	GRMZM2G104843	*Ts1_NP1*	*chr156‐m1*	W22	Loss	100 72.2	3′ end 5′ end	M	
*PhyC2*	GRMZM2G129889	*PhyC2_NP1*	*chr156‐m1*	W22	Loss	89.5	5′ end	M	
*Bt1*	GRMZM2G144081	*Bt1_NP1*	*chr156‐m1*	W22	Loss	43.2 45.7	3′ end 5′ end	M	
*Sam2*	GRMZM2G154397	*Sam2_NP1*	*chr156‐m1*	W22	Loss	79.5	3′ end	M	
*Mads1*	GRMZM2G171365	*Mads1_NP1*	*chr156‐m1*	W22	Loss	43.2	3′ end	W	1
*Lhcb1*	GRMZM2G351977	*Lhcb1_NP1*	*chr156‐m1*	W22	Loss	NA^b^	NA^b^	M	1
*Lhcb1*	GRMZM2G351977	*Lhcb1_NP2*	*chr156‐m1*	W22	Loss	NA^b^	NA^b^	M	
*Pl1*	GRMZM2G701063	*Pl1_NP1*	*chr156‐m1*	W22	Loss	NA^b^	NA^b^	M	

The names and IDs of maize genes with demonstrated nucleosome position (NP) change in one or more genotypes are listed. The genotypes within which NP differences were detected, along with the type of NP change (gain–gain of a positioned nucleosome or loss–loss of a positioned nucleosome) in each genotype, are indicated. For the NP change names, at loci with multiple NP change regions, the more 5′ region is designated as NP1 and the more 3′ region is designated as NP2. The percentage of methylation of CHH islands (% mCHHi) and the location of the CHHi relative to the genes is indicated. Similarity of NP in mutant (M) and wild type (W) to Nucleosome Occupancy Likelihood (NOL) was determined using comparison of the Pearson correlation coefficients. The genotypes more correlated with NOL are indicated as well as the number of Motif‐1 occurrences.

a Data from (Li et al., 2015).

b No data in (Li et al., 2015).

**Figure 4:**
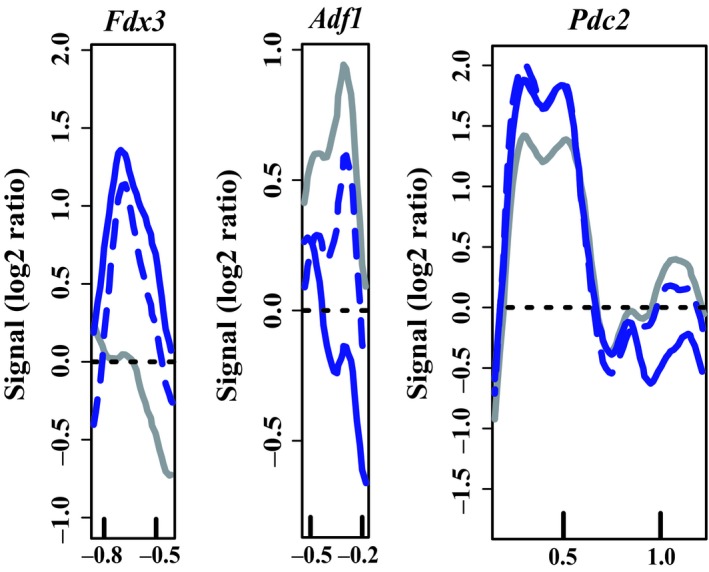
Nucleosome position changes in *chr101‐m1* and *chr101‐m3* mutants. Comparison of the nucleosome position in wild type B73 (solid gray line), homozygous *chr101‐m1* (solid blue line), and homozygous *chr101‐m3* (dashed blue line) plants. Genes are all oriented according to a left‐to‐right transcriptional direction, with the nucleotide position in kb relative to the transcription start site (0) indicated on the x‐axes. Relative fluorescent signals are indicated on the y‐axes (Signal (log2 ratio)). Three pools of three biological replicates were averaged for each plot, except for *chr101 m3*, which includes one pool of three biological replicates

The phylogenetic subgroup 1 proteins analyzed in this study were CHR101 and CHR106. Microarray analysis of nucleosome position in *chr101‐m1* compared to the wild‐type B73 genotype identified nucleosome position changes at the 3000‐bp region around the TSS of three different genes, including two nucleosome position changes near *Pdc2* (Figure 4; Table 3). Changes in nucleosome position were designated according to the locus name and nucleosome position change number; for example, *Adf1_NP1* refers to the first detected change at the *Adf1* locus. At the *Adf1_NP1* and *Pdc2_NP2* regions, a positioned nucleosome was lost in homozygous mutant plants, suggesting that a well‐positioned nucleosome had been removed. On the other hand, the gain of a positioned nucleosome was demonstrated in homozygous mutant plants at *Fdx3_NP1* and *Pdc2_NP1* region, suggesting that a well‐positioned nucleosome has been added. It is possible that the two changes at *Pdc2* actually reflect a shifted nucleosome from one region to another rather than an unrelated loss at one region and gain at another, consistent with the Arabidopsis DDM1 moving nucleosomes rather than removing them (Brzeski & Jerzmanowski, 2003). For each of these loci, qualitatively similar changes were observed in plants homozygous for either *chr101‐m1* or *chr101‐m3* (Figure 4), which supports the notion that this nucleosome position phenotype is associated with loss of CHR101 activity. Where there is a quantitative difference, *chr101‐m3* is always more similar to wild type than *chr101‐m1* although the *chr101‐m3* mutation is more likely to disrupt the protein coding capacity of *Chr101*.

**Figure 5:**
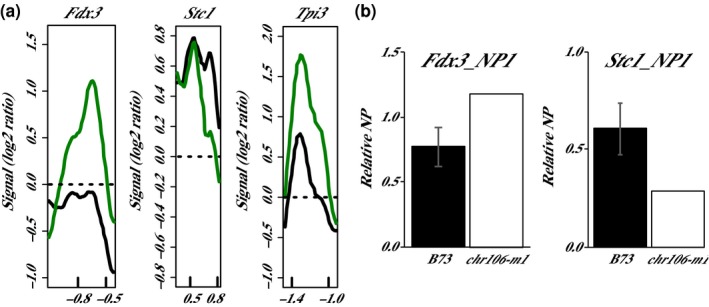
Nucleosome position (NP) changes in *chr106‐T11* and *chr106‐m1* mutants. (a) Comparison of the nucleosome position in wild‐type W22 plants (black line) and plants homozygous for *chr106‐T11* (green line). Genes are all oriented according to a left‐to‐right transcriptional direction, with the nucleotide position in kb relative to the transcription start site (0) indicated on the x‐axes. Relative fluorescent signals are indicated on the y‐axes (Signal (log2 ratio)). Three pools of three biological replicates were averaged for each plot. (b) Relative 2^‐∆∆Ct^ values (Relative NP) for wild type B73 (black bar) and homozygous *chr106‐m1* (white bar) plants at *Fdx3_NP1* and *Stc1_NP1*. The data for B73 represent averages ±*SD* (error bars) of three pools of three biological replicates and for *chr106‐m1* one pool of three biological replicates

The microarray analysis of *chr106‐T11* compared to the wild‐type W22 genotype identified three loci with altered nucleosome positioning. At *Stc1_NP1*, the loss of a positioned nucleosome was observed in homozygous mutant plants, suggesting nucleosome removal or relocation at these loci. At *Fdx3_NP1* and *Tpi3_NP1,* the gain of a positioned nucleosome was demonstrated in *chr106‐T11* homozygous plants compared to wild type (Figure 5a; Table 3) indicating the acquisition of a well‐positioned nucleosome. Due to the small number of loci with differences in chr106‐T11 homozygous plants, *chr106‐m1* homozygous plants were tested by focused qPCR of micrococcal nuclease (MNase)‐digested samples at *Fdx3_NP1* and *Stc1_NP1* rather than microarray. The *Tpi3_NP1* region was not amenable to qPCR analysis. Although statistical significance was not demonstrated, the nucleosome position changes observed in *chr106‐T11* at *Fdx3_NP1* and *Stc1_NP1* were also observed in *chr106‐m1* (Figure 5b), suggesting that the nucleosome position changes at *Fdx3_NP1* and *Stc1_NP1* are associated with the loss of CHR106 activity.

**Figure 6:**
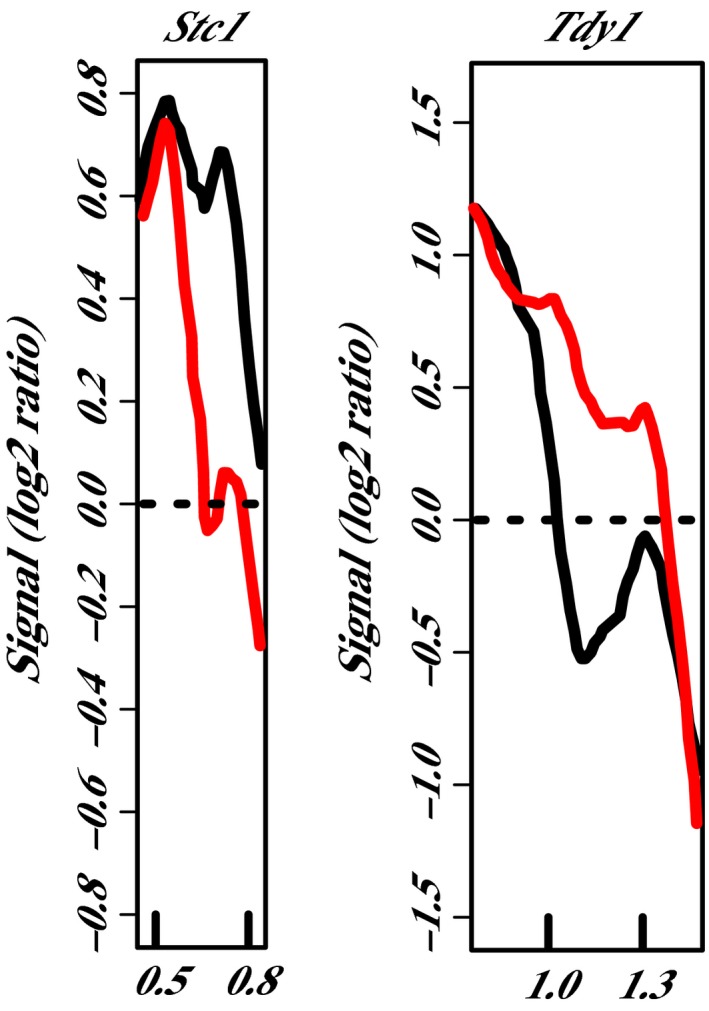
Nucleosome position changes in *chr127‐m1* mutants. Comparison of the nucleosome position in wild type W22 (black line) and homozygous *chr127‐m1* (red line) plants. Genes are all oriented according to a left‐to‐right transcriptional direction, with the nucleotide position in kb relative to the transcription start site (0) indicated on the x‐axes. Relative fluorescent signals are indicated on the y‐axes (Signal (log2 ratio)). Three pools of three biological replicates were averaged for each plot

**Figure 7:**
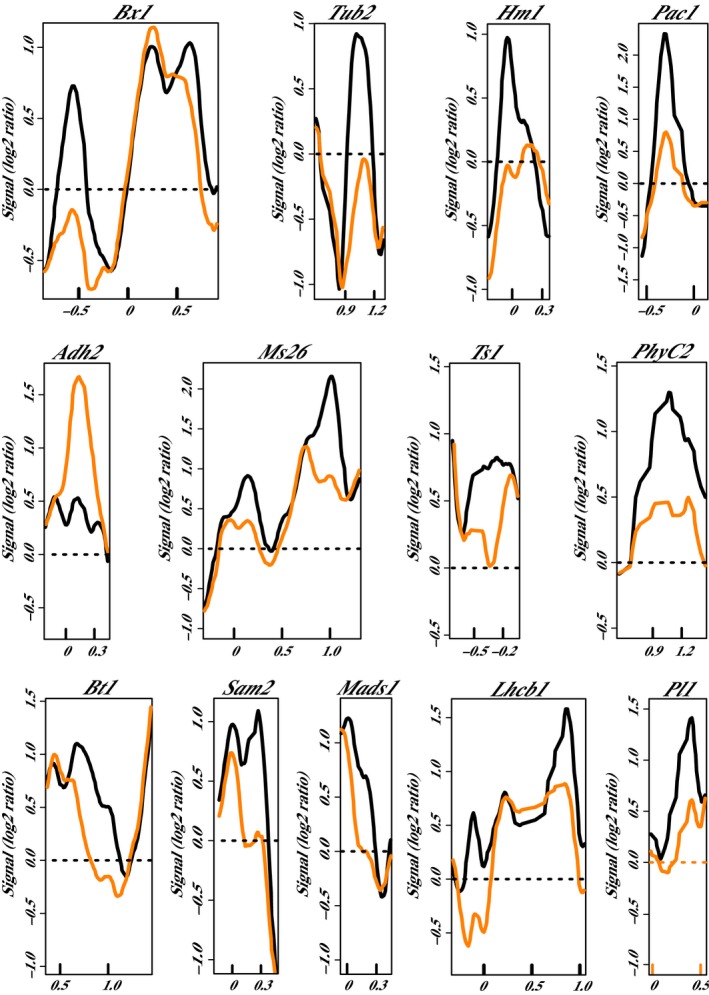
Nucleosome position changes in *chr156‐m1* mutants. Comparison of the nucleosome position in wild type W22 (black line) and homozygous *chr156‐m1* (orange line) plants. Genes are all oriented according to a left‐to‐right transcriptional direction, with the nucleotide position in kb relative to the transcription start site (0) indicated on the x‐axes. Relative fluorescent signals are indicated on the y‐axes (Signal (log2 ratio)). Three pools of three biological replicates were averaged for each plot

The phylogenetic subgroup 2 proteins analyzed in this study were RMR1, CHR127, and CHR156. In *chr127‐m1*, changes in nucleosome positioning were identified for two loci (Table 3). A positioned nucleosome was lost at *Stc1_NP1* in *chr127‐m1* and gained at *Tdy1_NP1* (Figure 6). Although the microarray was initially designed to study the 5′ end of genes, the small size of the *Tdy1* gene (819 bp) made it possible to include the entire gene in the 3000‐bp region of study, and the nucleosome position change observed at this locus in *chr127‐m1* was detected in the 3′UTR region, suggesting that the nucleosome position changes in this genotype were not limited to transcription start sites. Nucleosome position changes were detected for 13 genes in plants homozygous for *chr156‐m1*. Of these 13 genes, three had multiple nucleosome position changes, which contributed to a total of 16 discrete nucleosome position changes detected in this genotype (Figure 7; Table 3). In the homozygous *chr156‐m1* plants, positioned nucleosome was gained at *Adh2_NP1* compared to wild type, while it was lost at the other 15 regions.

Analysis of plants homozygous for *rmr1‐1* did not reveal any loci with significantly altered nucleosome positions compared to wild type, although two genes, *b1* and *pl1* (Hale et al., 2007) that have been shown to have higher levels of transcript abundance in *rmr1‐1,* were included on the microarray.

### 3.5 Similar nucleosome position changes were detected in multiple genotypes at the same loci

Analysis of nucleosome positioning identified some of the same loci with nucleosome position changes in different genotypes. *Tpi3_NP1* changes occurred between the B73 and W22 haplotypes (Fig. S3a), and between *chr106‐T11* and its wild type W22. While the gene expression of *Chr106* was reduced in W22 compared to B73 (Figure 2b), the lowest level of *Chr106* gene expression was observed in *chr106‐T11* (Figure 2a). At *Tpi3_NP1,* the most positioned nucleosome was in B73 and the least positioned nucleosome was in W22 (Fig. S3a), and homozygous *chr106‐T11* plants had an intermediate level of nucleosome positioning between that of B73 and W22. Nucleosome position changes were also shared between *chr101‐m1*,* chr101‐m3*, and *chr106‐T11* at *Fdx3_NP1* (Fig. S3b). This type of change could be indicative of overlapping or cooperative function between homologous proteins.

One locus also had phenotypes in plants deficient for both phylogenetic subgroups 1 and 2 proteins. A positioned nucleosome was lost at *Stc1_NP1* in *chr127‐m1* in a similar manner as observed in *chr106‐T11* (Fig. S3c). Common nucleosome position changes in multiple genotypes could indicate that nucleosome position at these loci is particularly susceptible to perturbation or that nucleosome position at these loci requires the coordinated action of multiple proteins or regulatory pathways. CHR127 has been shown to interact with PolV (Haag et al., 2014), while *chr106‐T11 *has published DNA methylation phenotypes (Li et al., 2014). Transcriptional gene silencing pathways have been indicated to utilize both chromatin proteins and plant‐specific polymerases to alter DNA methylation (reviewed by Wendte & Pikaard, 2016). One possible mechanism that could explain the shared nucleosome position changes at *Stc1_NP1* might require CHR127 for chromatin remodeling to allow PolV access to regulated loci and CHR106 for the coordinated alteration of DNA methylation at loci with PolV transcription.

### 3.6 CHH islands are highly methylated near loci with altered nucleosome positions in maize mutants with altered gene expression of chromatin proteins

In maize, RNA‐directed DNA methylation (RdDM) pathway‐dependent short sequences rich in asymmetric cytosine methylation (CHH) islands have been identified between some genes and transposable elements (TEs) and have been suggested to function as barriers between euchromatic and heterochromatic regions of the genome (Gent et al., 2013; Li et al., 2015). Loci with altered nucleosome positions in homozygous *chr101‐m1*,* chr106‐T11*,* chr127‐m1*, and *chr156‐m1* mutants were analyzed for proximity to CHH islands using published data (Li et al., 2015). Although a genomewide analysis showed that only 57% of CHH islands have a 20% or higher methylation level (Li et al., 2014), all CHH islands identified at loci with differential nucleosome position in the mutant genotypes of this study had methylation levels above 20% in B73 wild type (Table 3).

### 3.7 Nucleosome position correlates more with intrinsic sequence signals in wild‐type plants than in *chr101‐m1* and *chr127‐m1*

One important determinant of nucleosome position lies in the intrinsic properties of the DNA itself (reviewed in Fincher & Dennis, 2011). Nucleosome mapping microarray experiments have been used to computationally generate nucleosomal predictions, termed nucleosome occupancy likelihood (NOL) for the maize B73 genome (Fincher et al., 2013). Because DNA sequence information is the only input variable for this analysis, the predicted positions of nucleosomes generated by the NOL are related to the primary sequence of the analyzed loci. Chromatin remodelers are thought to override the sequence‐dependent positioning of nucleosomes, making comparisons with NOL useful in estimating whether nucleosome position is sequence‐ or protein‐directed.

The Pearson correlation coefficient was used to compare nucleosome position of the wild haplotypes and homozygous mutants to NOL. For all four of the loci with changes in nucleosome position in the *chr101‐m1* mutants, the NOL had a higher correlation with the position detected in the mutant genotype (Table 3). This is consistent with sequence‐dependent nucleosome positioning in genotypes with reduced levels of CHR101. Similarly, for two of the three nucleosome position change regions in the *chr106‐T11* genotypes, the plants homozygous for the mutant allele had a higher correlation to NOL, suggesting that in the wild‐type plants these nucleosomes are positioned away from sequence signals (Table 3).

For the two loci with changes in nucleosome position in the *chr127‐m1* homozygous plants, the NOL also had a higher Pearson correlation with the position detected in the mutants (Table 3), indicating a sequence‐dependent positioning of nucleosomes in the *chr127‐m1* mutant. Notably, *Chr127* gene expression is higher in homozygous *chr127‐m1* plants, which could result in *Chr127* overexpression rather than loss of function. The changes in nucleosome position in plants homozygous for *chr127‐m1* suggest that it may be a loss‐of‐function allele in spite of the elevated *Chr127* transcript levels detected by qRT‐PCR.

The most nucleosome position changes were identified in *chr156‐m1,* and at these loci, 13 of the 16 regions (81%) had a higher correlation between mutant and NOL (Table 3), suggesting protein‐directed nucleosome position in wild‐type plants. The other three regions indicated a higher correlation between wild type and NOL suggesting distinct types of loci‐dependent effects in this genotype.

### 3.8 Nucleosome position at some loci is similar in *chr127‐m1* and *mop2‐1*

**Figure 8:**
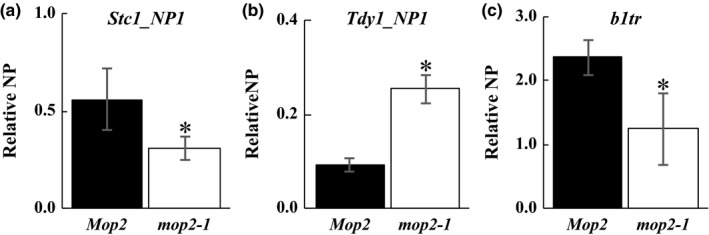
MNase‐qPCR analysis of nucleosome position (NP) in homozygous *mop2‐1* plants. Comparison of relative 2^−∆∆Ct^ values (Relative NP) for wild type *Mop2* (black bar) and homozygous *mop2‐1* (white bar) plants at *Stc1_NP1* (a), *Tdy1_NP1* (b), and *b1tr* (c). The data represent averages ±*SD* (error bars) of three pools of three biological replicates. Student's *t* test (*p* ≤ .05) was used to identify statistically significant changes between mutant and WT (*)

Because CHR127 is known to interact in a PolV complex with other proteins including mediator of paramutation2 (MOP2) (Haag et al., 2014), it seemed plausible that CHR127 might function in a PolV‐mediated regulatory pathway. Thus, CHR127‐mediated changes in nucleosome position might also be dependent upon MOP2 function. Nucleosome position was assayed in *mop2‐1* homozygous plants at *Stc1_NP1* and *Tdy1_NP1,* where nucleosome position phenotypes had been noted in *chr127‐m1*. The nucleosome position at *Stc1_NP1* and at *Tdy1_NP1* showed similar changes in *mop2‐1* (Figure 8a,b) as observed in *chr127‐m1* homozygous plants (Figure 6).

While MOP2 is the second largest subunit of PolIV and PolV (Sidorenko et al., 2009), mediator of paramutation1 (MOP1) appears to interact only with PolIV (Haag et al., 2014). Previous studies have analyzed the nucleosome position of the homozygous *mop1‐1* mutant plants and wild‐type siblings, and the nucleosome position changes observed for *mop1‐1* (Labonne et al., 2013) were not observed in *chr127‐m1*. An additional statistical analysis (Student's *t* test) confirmed that nucleosome position changes observed in both *mop2‐1/mop2‐1* and *chr127‐m1/chr127‐m1* plants were not detectable in *mop1‐1/mop1‐1* plants (data not shown). These results indicate that nucleosome position at *Stc1* and *Tdy1* is likely associated with a PolV complex that includes MOP2 and CHR127 rather than a PolIV complex that would also include MOP1.

### 3.9 A positioned nucleosome is lost in *mop2‐1* at the *b1 tandem repeats*

MOP2 and other genetically interacting proteins are required for appropriate transcriptional regulation of the *b1* gene (Alleman et al., 2006; Belele et al., 2013; Hale et al., 2007; Hollick et al., 2005; Sidorenko et al., 2009; Sloan, Sidorenko, & McGinnis, 2014). Two epialleles of the *b1* gene, *B‐I* (high expressing) and *B'* (low expressing), are associated with distinct chromatin structure at a regulatory region 100‐kb upstream of the *b1* gene (Haring et al., 2010; Louwers et al., 2009; Stam, Belele, Dorweiler, & Chandler, 2002), and changes in gene expression of *b1* in different tissues have been shown to be associated with changes in nucleosome position in these regulatory regions (Haring et al., 2010). To determine whether MOP2‐dependent changes in the gene expression of *b1* are associated with changes in nucleosome position, MNase‐qPCR was performed in *mop2‐1* homozygous mutants at the distal *b1 tandem repeats* (*b1tr*) with characterized chromatin structural changes (Haring et al., 2010). Nucleosome position was compared in *Mop2 B'* wild type, and *mop2‐1* homozygous mutant plants with upregulated *B'*, in a W23/K55 background. A positioned nucleosome was lost in the *mop2‐1* homozygous plants compared to wild‐type plants (Figure 8c), suggesting that reduced MOP2 function is associated with changes in nucleosome position at the *b1* regulatory regions. Because PolV regulation of *b1* is allele‐specific (Stam, Belele, Dorweiler, et al., 2002) and most genotypes do not have the appropriate *b1* allele for the analysis of *b1* gene expression and chromatin structure, the effects of the other predicted chromatin proteins on *b1tr* were not tested, and no nucleosome position difference was detected at the 3000‐bp region around the TSS of the *b1* gene on the microarray (data not shown).

### 3.10 Gene expression of *Stc1* is reduced in both the *chr127‐m1* mutants and in the loss‐of‐function *mop2‐1* mutants

**Figure 9:**
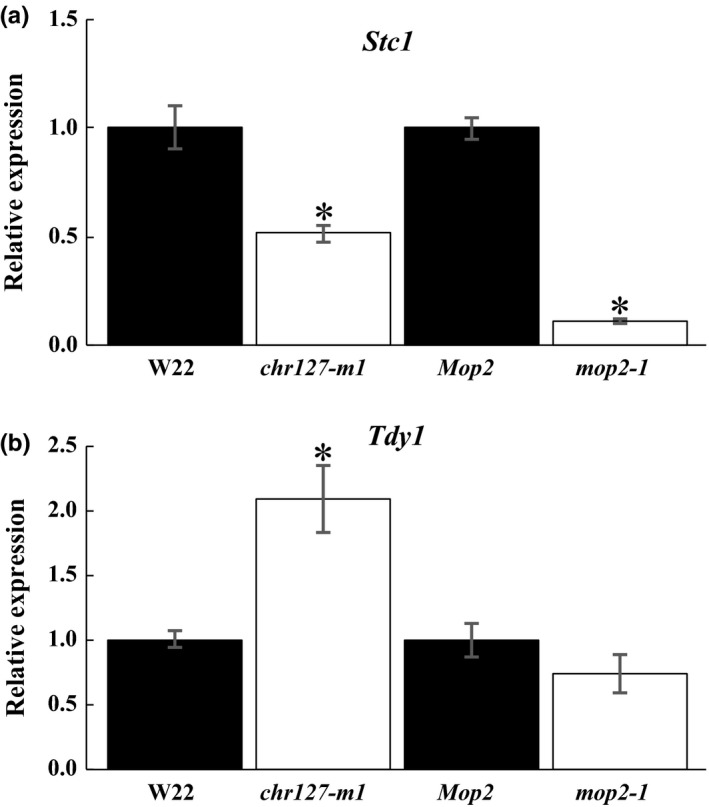
Gene expression of *Stc1* and *Tdy1* in homozygous *chr127‐m1* and *mop2‐1* plants. Relative expression levels of *Stc1* (a) and *Tdy1* (b) were determined by qRT‐PCR and calculated as 2−^∆∆Ct^. Gene expression was determined in wild‐type (black bar) and homozygous mutant (white bar) individuals. The data represent averages ±*SD* (error bars) of three pools of three biological replicates that were normalized to 45S rRNA. Student's *t* test (*p* ≤ .05) was used to identify statistically significant changes between mutant and WT (*)

The nucleosome position changes at *b1tr* in *mop2‐1* homozygous plants (Figure 8c) were similar to those observed in the wild‐type plant tissues where *B‐I* gene expression is highest (Haring et al., 2010), suggesting that the PolV‐MOP2 regulatory pathway coordinately influences gene expression and nucleosome position. To determine whether MOP2‐ and CHR127‐associated nucleosome positions at *Stc1_NP1* and *Tdy1_NP1* are also associated with specific gene expression levels, qRT‐PCR analysis of transcript abundance was performed. The loss of a positioned nucleosome downstream of the TSS at *Stc1_NP1* in *chr127‐m1* (Figure 6) and *mop2‐1* (Figure 8a) was associated with significantly reduced gene expression of *Stc1* in both *chr127‐m1* and *mop2‐1* compared to their respective wild types (Figure 9a). The gain of a positioned nucleosome in the 3′UTR at *Tdy1_NP1* in *chr127‐m1* (Figure 6) and *mop2‐1* (Figure 8b) was associated with significantly increased gene expression of *Tdy1* in *chr127‐m1,* but there was no significant change in *mop2‐1* (Figure 9b), even though the nucleosome position phenotype was observed in both the *chr127‐m1* and *mop2‐1* mutants (Figures 6 and 8b). Thus, nucleosome position and gene expression changes were not always observed together and not always in a consistent manner with the loss of silencing effect observed at *b1* in *mop2‐1* homozygous plants.

### 3.11 Nucleosome position changes are associated with changes in gene expression in *chr156‐m1* mutants, but the changes do not occur in *mop2‐1* mutants

**Figure 10:**
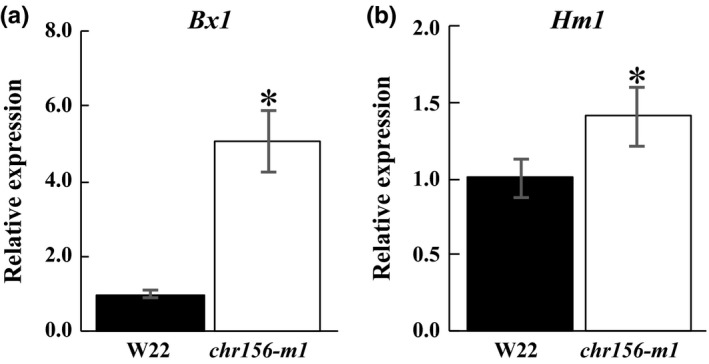
Gene expression of *Bx1* and *Hm1* in *chr156‐m1*. Relative expression levels of *Bx1* (a) and *Hm1* (b) were determined by qRT‐PCR and calculated as 2^−∆∆Ct^. Gene expression was determined in homozygous wild type (W22, black bar) and homozygous *chr156‐m1* (white bar) plants. The data represent averages ±*SD* (error bars) of three pools of three biological replicates that were normalized to 45S rRNA. Student's *t* test (*p* ≤ .05) was used to identify statistically significant changes between mutant and WT (*)

CHR127 and CHR156 have 86% sequence identity, but only CHR127 seems to interact with PolV (Haag et al., 2014). Two loci with differential nucleosome positioning in *chr156‐m1* (*Hm1_NP1* and *Bx1_NP1*) were also tested for nucleosome position change in plants homozygous for *mop2‐1*. Neither of the loci had a significant nucleosome position change in *mop2‐1* (data not shown), suggesting that nucleosome position phenotypes are shared between *mop2‐1* and *chr127‐m1*, but not *mop2‐1* and *chr156‐m1*. The loss of positioned nucleosomes in homozygous *chr156‐m1* plants (Figure 7) was associated with increased gene expression of *Bx1* and *Hm1* as determined by qRT‐PCR (Figure 10a,b).

### 3.12 The plant‐specific C2C2‐D of transcription factor binding motif is present at several nucleosome position change regions and at the *b1 tandem repeats*

The nucleosome position changes in multiple genotypes and comparisons with NOL suggested that DNA sequence was a relevant factor in nucleosome position at the tested loci. To determine whether different chromatin proteins target specific DNA sequences, loci with differential nucleosome positions were analyzed for DNA sequence motifs using the MEME application (Bailey & Elkan, 1994) of the MEME Suite (Bailey et al., 2009). This motif search application requires a background Markov model. As the molecular analysis by microarray was TSS focused, the Markov model used in the analysis was generated from the combined TSS sequences represented on the microarray.

**Figure 11:**
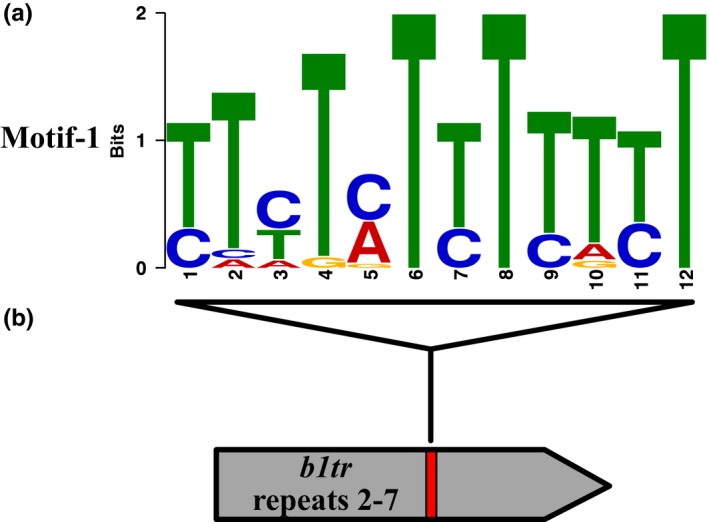
Motif‐1 enriched in loci with differential nucleosome position. (a) Diagram of the sequence of Motif‐1. (b) The location of Motif‐1 (red box) along repeats 2 through 7 of the *b1 tandem repeat*s (*b1tr*) as determined by FIMO. Diagram is to scale with Motif‐1 representing 12 nucleotides

Through microarray analysis of nine genotypes, a total of 30 distinct loci with nucleosome position changes in any genotype were detected. All 30 loci were subjected to a group analysis by MEME using an E‐value of 0.05 as a threshold to identify motifs. One motif (Motif‐1) was identified at nine loci (Figure 11a; Tables 2 and 3). As seven of these loci are those with altered nucleosome position in the mutants of phylogenetic subgroup 2 proteins, Motif‐1 seems to have some specificity toward the putative chromatin proteins that belong to phylogenetic subgroup 2.

Because MOP2 and CHR127 appear to have some overlapping function and Motif‐1 was present at *Tdy1_NP1*, Motif‐1 was compared with the regulatory sequence of *b1,* which is a series of tandem repeats (Stam, Belele, Dorweiler, et al., 2002). Analysis of Motif‐1 against *b1tr* using FIMO (Grant et al., 2011) of the MEME Suite identified a significant (*q*‐value ≤0.05) match. This match was present in six of the seven tandem repeats of *b1tr* (Figure 11b), which are associated with transcriptional regulation of *b1* (Stam, Belele, Dorweiler, et al., 2002).

Although SWI/SNF chromatin remodelers interact with and modify chromatin, direct DNA interaction has not been demonstrated, and many chromatin remodelers have been shown to be recruited by transcription factors (reviewed by Kwon & Wagner, 2007). To determine whether Motif‐1 is a transcription factor recognition motif, it was compared to previously identified maize and Arabidopsis transcription factor motifs (O'Malley et al., 2016; Yu et al., 2015) using the MEME Suite's motif comparison tool (TOMTOM, Gupta et al., 2007). The most similar previously identified maize putative transcription factor binding site was ID0180 (*E*‐value ≤0.05), although very little information is available about the biological function of ID0180. The comparison to Arabidopsis revealed that the most similar previously identified motif is the plant‐specific C2C2‐D of transcription factor recognition motif (*E*‐value ≤0.05). These results could suggest that the chromatin proteins of this study and a C2C2‐Dof‐like transcription factor may act at Motif‐1 sequences to modify nucleosome position and facilitate transcriptional regulation. It can also be suggested that the motif corresponding to ID0180 could be a C2C2‐Dof transcription factor recognition sequence.

## 4 DISCUSSION

Using a microarray‐based nucleosome mapping approach, changes in nucleosome positioning were detected in genotypes with mutant alleles for four predicted chromatin‐related proteins (CHR101, CHR106, CHR127, and CHR156). All of the chromatin proteins tested in this study are homologous with known members of SWI/SNF complexes in other species. The majority of the loci with nucleosome position changes in homozygous mutants showed evidence of nucleosomes being moved or held away from sequence‐dependent positions by functional chromatin proteins. These results are consistent with the human hSWI/SNF proteins, which move nucleosomes away from their sequence‐favored positions (Sims, Baughman, & Schnitzler, 2008; Sims, Lane, Ulyanova, & Schnitzler, 2007), and suggest a role for nucleosome positioning for all the maize chromatin proteins analyzed in this study, except potentially RMR1.

Interestingly, although plants homozygous for *rmr1‐1* have phenotypes related to the transcriptional regulation of gene expression, suggesting that some functionality is disrupted by this mutation, no changes in nucleosome position were observed in plants homozygous for *rmr1‐1*. This could be because RMR1 does not function to modify or maintain nucleosome positioning, because RMR1 regulates loci other than the ones investigated, or because this particular allele of RMR1 does not disrupt the specific function of RMR1 in nucleosome positioning. RMR1 possesses the domains common to chromatin remodelers, including a Helicase_C domain, but it is possible that RMR1‐dependent regulatory mechanisms do not involve changes in nucleosome position and these domains instead support functions other than chromatin remodeling, like resolving the double helix during transcription. In support of this, two genes known to be transcriptionally regulated by RMR1 (Hale et al., 2007; Hollick & Chandler, 2001) were on the microarray and did not exhibit differential nucleosome position in *rmr1‐1* homozygous plants. It is also possible that the *rmr1‐1* mutation may not be sufficient to impede RMR1‐mediated nucleosome positioning, although the phenotypes associated with *rmr1‐1* suggest that this is a deleterious allele. It is also possible that analysis of additional tissues, developmental stages, or regions not on the microarray may reveal RMR1‐dependent nucleosome position phenotypes.

Consistent with prior comparisons of gene expression between maize haplotypes (Dong et al., 2016), this analysis revealed differential gene expression of *Chr106* between B73 and W22. This study also identified nucleosome position changes between B73 and W22 in regions with minimal sequence polymorphisms. Shared nucleosome position changes between the two haplotypes and in the homozygous *chr106‐T11* mutants, and reduced *Chr106* expression in W22 suggests that differential gene expression of chromatin proteins may influence haplotype‐specific phenotypes.

CHR101 and CHR106 are highly similar to one another, and this analysis detected both shared and distinct changes in nucleosome position in *chr101* and *chr106* genotypes. Previous research has demonstrated the lethality of double mutants and some phenotypic differences in genomewide DNA methylation between the *chr101* and *chr106* genotypes (Li et al., 2014), suggesting that CHR101 and CHR106 may have some distinct roles. Collectively, this suggests that while CHR101 and CHR106 might coordinate activities at some loci, they may also have distinct and important locus‐specific functions.

Sequence similarity with a known Arabidopsis transcription factor binding motif suggests that a C2C2‐Dof type transcription factor may interact with some loci with changes in nucleosome positioning. Motif‐1 was detected in *Fdx3_NP1*,* Tdy1_NP1*,* Bx1_NP2*,* Hm1_NP1*,* Pac1_NP1*,* Adh2_NP1*,* Lhcb1_NP1*,* Mads1_NP1*, and *Gpc1_NP1*, which seem to have nucleosome position regulated by CHR101, CHR106, CHR127, and CHR156. This may indicate that a C2C2‐Dof type transcription factor recruits these chromatin proteins to regulated loci.

It has been proposed that RdDM activity at CHH islands maintains silencing of transposable elements by forming an epigenetic barrier that blocks transcription from adjacent genes from spreading into the transposable elements (Li et al., 2015). This would mean that loss of RdDM might not change gene expression, although maize mutants thought to be defective in RdDM have demonstrated changes in gene expression of *b1* (Alleman et al., 2006; Belele et al., 2013; Hale et al., 2007; Hollick et al., 2005; Sidorenko et al., 2009; Sloan et al., 2014) and other genes (Jia et al., 2009). The fact that some nucleosome position changes were associated with changes in gene expression and some were not, combined with the presence of CHH islands near some nucleosome position change regions, suggests that RdDM, nucleosome position, and gene expression may intersect in distinct ways at different loci. Although a change in one characteristic is not consistently correlated with a change in the others, these characteristics frequently overlap at apparently regulated loci.

The *chr127‐m1* allele used in this study is associated with increased gene expression of *Chr127* compared to wild type, while *mop2‐1* homozygous plants are loss‐of‐function mutants (Sidorenko et al., 2009). Transposon insertions in the 5′UTR with elevated gene expression are sometimes associated with loss‐of‐function phenotypes such as hypomethylation (Prelich, 2012; Robbins et al., 2008), but could also lead to phenotypes caused by overexpression of a functional protein. The fact that homozygous *chr127‐m1* plants share the same phenotype as plants homozygous for the loss‐of‐function *mop2‐1* alleles suggests that the transposon insertion in *chr127‐m1* also results in loss of function.

This work has demonstrated altered nucleosome positions in several maize genotypes, including inbred haplotypes and mutant genotypes. Nucleosome position changes were observed at genes encoding proteins with a range of predicted biological functions. Collectively, these results suggest that nucleosome position may contribute to the phenotypic diversity observed in maize haplotypes and may be useful in exploiting and understanding this important crop plant. MNase‐sequencing approaches using these and other mutant genotypes would likely identify additional loci that are impacted by these mechanisms.

## AUTHOR CONTRIBUTIONS

K.M.M. and L.K.S. designed the experiments. L.K.S. performed the experiments. K.M.M. and L.K.S. interpreted the data and wrote the article.

## References

[pld319-bib-0001] Alexeev, A. , Mazin, A. , & Kowalczykowski, S. C. (2003). Rad54 protein possesses chromatin‐remodeling activity stimulated by the Rad51‐ssDNA nucleoprotein filament. Natural Structural Biology, 10, 182–186. 10.1038/nsb901 12577053

[pld319-bib-0002] Alleman, M. , Sidorenko, L. , McGinnis, K. , Seshadri, V. , Dorweiler, J. E. , White, J. , … Chandler, V. L. (2006). An RNA‐dependent RNA polymerase is required for paramutation in maize. Nature, 442, 295–298. 16855589 10.1038/nature04884

[pld319-bib-0003] Altschul, S. , Gish, W. , Miller, W. , Myers, E. , & Lipman, D. (1990). Basic local alignment search tool. Journal of Molecular Biology, 215, 403–410. 2231712 10.1016/S0022-2836(05)80360-2

[pld319-bib-0004] Bailey, T. , Bodén, M. , Buske, F. , Frith, M. , Grant, C. , Clementi, L. , … Noble, W. (2009). MEME SUITE: Tools for motif discovery and searching. Nucleic Acids Research, 37, 202–208. 10.1093/nar/gkp335 PMC2703892 19458158

[pld319-bib-0005] Bailey, T. , & Elkan, C. (1994). Fitting a mixture model by expectation maximization to discover motifs in biopolymers Proceedings of the second international conference on intelligent systems for molecular biology (pp. 28–36). Menlo Park, California: AAAI Press. 7584402

[pld319-bib-0006] Barkan, A. , & Martienssen, R. A. (1991). Inactivation of maize transposon Mu suppresses a mutant phenotype by activating an outward‐reading promoter near the end of Mu1. PNAS, 88, 3502–3506. 1849660 10.1073/pnas.88.8.3502 PMC51476

[pld319-bib-0007] Belele, C. L. , Sidorenko, L. , Stam, M. , Bader, R. , Arteaga‐Vazquez, M. A. , & Chandler, V. L. (2013). Specific tandem repeats are sufficient for paramutation‐induced trans‐generational silencing. PLoS Genetics, 9(10), e1003773. 24146624 10.1371/journal.pgen.1003773 PMC3798267

[pld319-bib-0008] Brzeski, J. , & Jerzmanowski, A. (2003). Deficient in DNA methylation 1 (DDM1) defines a novel family of chromatin‐remodeling factors. Journal of Biological Chemistry, 278, 823–828. 12403775 10.1074/jbc.M209260200

[pld319-bib-0009] Clapier, C. R. , & Cairns, B. R. (2009). The biology of chromatin remodeling complexes. Annual Review of Biochemistry, 78, 273–304. 10.1146/annurev.biochem.77.062706.153223 19355820

[pld319-bib-0010] Dong, J. , Feng, Y. , Kumar, D. , Zhang, W. , Zhu, T. , Luo, M.‐C. , & Messing, J. (2016). Analysis of tandem gene copies in maize chromosomal regions reconstructed from long sequence reads. PNAS, 113, 7949–7956. 27354512 10.1073/pnas.1608775113 PMC4961126

[pld319-bib-0011] Dorweiler, J. E. , Carey, C. C. , Kubo, K. M. , Hollick, J. B. , Kermicle, J. L. , & Chandler, V. L. (2000). Mediator of paramutation1 is required for establishment and maintenance of paramutation at multiple maize loci. Plant Cell, 12, 2101–2118. 11090212 10.1105/tpc.12.11.2101 PMC150161

[pld319-bib-0012] Druliner, B. R. , Fincher, J. A. , Sexton, B. S. , Vera, D. L. , Roche, M. , Lyle, S. , & Dennis, J. H. (2013). Chromatin patterns associated with lung adenocarcinoma progression. Cell Cycle, 12, 1536–1543. 23598721 10.4161/cc.24664 PMC3680533

[pld319-bib-0013] Edgar, R. C. (2004). MUSCLE: Multiple sequence alignment with high accuracy and high throughput. Nucleic Acids Research, 32, 1792–1797. 15034147 10.1093/nar/gkh340 PMC390337

[pld319-bib-0014] Eisen, J. A. , Sweder, K. S. , & Hanawalt, P. C. (1995). Evolution of the SNF2 family of proteins : Subfamilies with distinct sequences and functions. Nucleic Acids Research, 23(14), 2715–2723. 7651832 10.1093/nar/23.14.2715 PMC307096

[pld319-bib-0015] Fincher, J. , & Dennis, J. (2011). DNA sequence contribution to nucleosome distribution In Craig J. & Wong N. (Eds.), Epigenetics: A reference manual (pp. 133–142). Norwich, UK: Horizon Scientific Press.

[pld319-bib-0016] Fincher, J. A. , Vera, D. L. , Hughes, D. D. , McGinnis, K. M. , Dennis, J. H. , & Bass, H. W. (2013). Genome‐wide prediction of nucleosome occupancy in maize reveals plant chromatin structural features at genes and other elements at multiple scales. Plant Physiology, 162, 1127–1141. 23572549 10.1104/pp.113.216432 PMC3668044

[pld319-bib-0017] Finn, R. D. , Coggill, P. , Eberhardt, R. Y. , Eddy, S. R. , Mistry, J. , Mitchell, A. L. , … Bateman, A. (2016). The Pfam protein families database: Towards a more sustainable future. Nucleic Acids Research, 44, D279–D285. 26673716 10.1093/nar/gkv1344 PMC4702930

[pld319-bib-0018] Flaus, A. , Martin, D. M. A. , Barton, G. J. , & Owen‐Hughes, T. (2006). Identification of multiple distinct Snf2 subfamilies with conserved structural motifs. Nucleic Acids Research, 34, 2887–2905. 16738128 10.1093/nar/gkl295 PMC1474054

[pld319-bib-0019] Gent, J. I. , Ellis, N. A. , Guo, L. , Harkess, A. E. , Yao, Y. , Zhang, X. , & Dawe, R. K. (2013). CHH islands: De novo DNA methylation in near‐gene chromatin regulation in maize. Genome, 23, 628–637. 10.1101/gr.146985.112 PMC3613580 23269663

[pld319-bib-0020] Grant, C. E. , Bailey, T. L. , & Noble, W. S. (2011). FIMO: Scanning for occurrences of a given motif. Bioinformatics, 27, 1017–1018. 21330290 10.1093/bioinformatics/btr064 PMC3065696

[pld319-bib-0021] Gupta, S. , Stamatoyannopolous, J. A. , Bailey, T. , & Noble, W. (2007). Quantifying similarity between motifs. Genome Biology, 8, 24. 10.1186/gb-2007-8-2-r24 PMC1852410 17324271

[pld319-bib-0022] Haag, J. R. , Brower‐Toland, B. , Krieger, E. K. , Sidorenko, L. , Nicora, C. D. , Norbeck, A. D. , … Pikaard, C. S. (2014). Functional diversification of maize RNA polymerase IV and V subtypes via alternative catalytic subunits. Cell Reports, 9, 378–390. 25284785 10.1016/j.celrep.2014.08.067 PMC4196699

[pld319-bib-0023] Hale, C. J. , Erhard, K. F. , Lisch, D. , & Hollick, J. B. (2009). Production and processing of siRNA precursor transcripts from the highly repetitive maize genome. PLoS Genetics, 5(8), e1000598. 19680464 10.1371/journal.pgen.1000598 PMC2725412

[pld319-bib-0024] Hale, C. J. , Stonaker, J. L. , Gross, S. M. , & Hollick, J. B. (2007). A novel Snf2 protein maintains trans‐generational regulatory states established by paramutation in maize. PLoS Biology, 5, e275. 17941719 10.1371/journal.pbio.0050275 PMC2020503

[pld319-bib-0025] Han, S.‐K. , Sang, Y. , Rodrigues, A. , Wu, M.‐F. , Rodriguez, P. L. , & Wagner, D. (2012). The SWI2/SNF2 chromatin remodeling ATPase BRAHMA represses abscisic acid responses in the absence of the stress stimulus in Arabidopsis. Plant Cell, 24, 4892–4906. 23209114 10.1105/tpc.112.105114 PMC3556964

[pld319-bib-0026] Haring, M. , Bader, R. , Louwers, M. , Schwabe, A. , Van Driel, R. , & Stam, M. (2010). The role of DNA methylation, nucleosome occupancy and histone modifications in paramutation. Plant Journal, 63, 366–378. 20444233 10.1111/j.1365-313X.2010.04245.x

[pld319-bib-0027] van Heerwaarden, J. , Hufford, M. B. , & Ross‐Ibarra, J. (2012). Historical genomics of North American maize. PNAS, 109, 12420–12425. 22802642 10.1073/pnas.1209275109 PMC3412004

[pld319-bib-0028] Hollick, J. , & Chandler, V. (2001). Genetic factors required to maintain repression of a paramutagenic maize pl1 allele. Genetics, 157(1), 369–378. 11139517 10.1093/genetics/157.1.369 PMC1461487

[pld319-bib-0029] Hollick, J. B. , Kermicle, J. L. , & Parkinson, S. E. (2005). Rmr6 maintains meiotic inheritance of paramutant states in Zea mays. Genetics, 171, 725–740. 16020780 10.1534/genetics.105.045260 PMC1456783

[pld319-bib-0030] Hollick, J. B. , Patterson, G. I. , Asmundsson, I. M. , & Chandler, V. L. (2000). Paramutation alters regulatory control of the maize pl locus. Genetics, 154, 1827–1838. 10747073 10.1093/genetics/154.4.1827 PMC1461010

[pld319-bib-0031] Ito, T. , Tarutani, Y. , To, T. K. , Kassam, M. , Duvernois‐Berthet, E. , Cortijo, S. , … Kakutani, T. (2015). Genome‐wide negative feedback drives transgenerational DNA methylation dynamics in *Arabidopsis* . PLoS Genetics, 11, 1–26. 10.1371/journal.pgen.1005154 PMC4406451 25902052

[pld319-bib-0032] Jeddeloh, J. A. , Bender, J. , & Richards, E. J. (1998). The DNA methylation locus DDM1 is required for maintenance of gene silencing in *Arabidopsis* . Genes & Development, 12, 1714–1725. 9620857 10.1101/gad.12.11.1714 PMC316876

[pld319-bib-0033] Jeddeloh, J. A. , Stokes, T. L. , & Richards, E. J. (1999). Maintenance of genomic methylation requires a SWI2/SNF2‐like protein. Nature Genetics, 22, 94–97. 10319870 10.1038/8803

[pld319-bib-0034] Jia, Y. , Lisch, D. R. , Ohtsu, K. , Scanlon, M. J. , Nettleton, D. , & Schnable, P. S. (2009). Loss of RNA‐dependent RNA polymerase 2 (RDR2) function causes widespread and unexpected changes in the expression of transposons, genes, and 24‐nt small RNAs. PLoS Genetics, 5, e1000737. 19936292 10.1371/journal.pgen.1000737 PMC2774947

[pld319-bib-0035] Jiang, C. , & Pugh, B. F. (2009). Nucleosome positioning and gene regulation: Advances through genomics. Nature Reviews Genetics, 10, 161–172. 10.1038/nrg2522 PMC4860946 19204718

[pld319-bib-0036] Jones, D. T. , Taylor, W. R. , & Thornton, J. M. (1992). The rapid generation of mutation data matrices from protein sequences. Computer Applications in the Biosciences, 8, 275–282. 1633570 10.1093/bioinformatics/8.3.275

[pld319-bib-0037] Kanno, T. , Mette, M. F. , Kreil, D. P. , Aufsatz, W. , Matzke, M. , & Matzke, A. J. M. (2004). Involvement of putative SNF2 chromatin remodeling protein DRD1 in RNA‐directed DNA methylation. Current Biology, 14, 801–805. 15120073 10.1016/j.cub.2004.04.037

[pld319-bib-0038] Kumar, S. , Stecher, G. , & Tamura, K. (2016). MEGA7: Molecular evolutionary genetics analysis version 7.0 for bigger datasets. Molecular Biology and Evolution, 33, msw054. 10.1093/molbev/msw054 PMC8210823 27004904

[pld319-bib-0039] Kwon, C. S. , & Wagner, D. (2007). Unwinding chromatin for development and growth: A few genes at a time. Trends in Genetics, 23, 403–412. 17566593 10.1016/j.tig.2007.05.010

[pld319-bib-0040] Labonne, J. D. J. , Dorweiler, J. E. , & McGinnis, K. M. (2013). Changes in nucleosome position at transcriptional start sites of specific genes in *Zea mays* mediator of paramutation1 mutants. Epigenetics, 8, 398–408. 23538550 10.4161/epi.24199 PMC3674049

[pld319-bib-0041] Laurent, B. C. , Treich, I. , & Carlson, M. (1993). The yeast SNF2/SWI2 protein has DNA‐ stimulated ATPase activity required for transcriptional activation. Genes & Development, 7, 583–591. 8458575 10.1101/gad.7.4.583

[pld319-bib-0042] Law, J. A. , Ausin, I. , Johnson, L. M. , Vashisht, A. A. , Zhu, J. , Wohlschlegel, A. , & Jacobsen, S. E. (2010). A protein complex required for polymerase V transcripts and RNA‐directed DNA methylation in plants. Current Biology, 20, 951–956. 20409711 10.1016/j.cub.2010.03.062 PMC2972704

[pld319-bib-0043] Li, Q. , Eichten, S. R. , Hermanson, P. J. , Zaunbrecher, V. M. , Song, J. , Wendt, J. , … Springer, N. M. (2014). Genetic perturbation of the maize methylome. Plant Cell, 26, 4602–4616. 25527708 10.1105/tpc.114.133140 PMC4311211

[pld319-bib-0044] Li, Q. , Gent, J. I. , Zynda, G. , Song, J. , Makarevitch, I. , Hirsch, C. D. , … Springer, N. M. (2015). RNA‐directed DNA methylation enforces boundaries between heterochromatin and euchromatin in the maize genome. PNAS, 112, 14728–14733. 26553984 10.1073/pnas.1514680112 PMC4664327

[pld319-bib-0045] Liu, K. , Goodman, M. , Muse, S. , Smith, J. S. , Buckler, E. , & Doebley, J. (2003). Genetic structure and diversity among maize inbred lines as inferred from DNA microsatellites. Genetics, 165, 2117–2128. 14704191 10.1093/genetics/165.4.2117 PMC1462894

[pld319-bib-0046] Livak, K. J. , & Schmittgen, T. D. (2001). Analysis of relative gene expression data using real‐time quantitative PCR and the 2^(‐∆∆CT)^ method. Methods, 25, 402–408. 11846609 10.1006/meth.2001.1262

[pld319-bib-0047] Louwers, M. , Bader, R. , Haring, M. , Van Driel, R. , De Laat, W. , & Stam, M. (2009). Tissue‐and expression level‐specific chromatin looping at maize b1 Epialleles. Plant Cell, 21, 832–842. 19336692 10.1105/tpc.108.064329 PMC2671708

[pld319-bib-0048] Madzima, T. F. , Mills, E. S. , Gardiner, J. M. , & McGinnis, K. M. (2011). Identification of epigenetic regulators of a transcriptionally silenced transgene in maize. G3, 1, 75–83. 22384320 10.1534/g3.111.000232 PMC3276119

[pld319-bib-0049] McCarty, D. R. , & Meeley, R. B. (2009). Transposon resources for forward and reverse genetics in maize In Bennetzen J. L. & Hake S. C. (Eds.), Handbook of maize: Genetics and genomics (pp. 561–584). Berlin: Springer.

[pld319-bib-0050] McGinnis, K. M. , Springer, C. , Lin, Y. , Carey, C. C. , & Chandler, V. (2006). Transcriptionally silenced transgenes in maize are activated by three mutations defective in paramutation. Genetics, 173, 1637–1647. 16702420 10.1534/genetics.106.058669 PMC1526669

[pld319-bib-0051] O'Malley, R. C. , Huang, S. S. C. , Song, L. , Lewsey, M. G. , Bartlett, A. , Nery, J. R. , … Ecker, J. R. (2016). Cistrome and epicistrome features shape the regulatory DNA landscape. Cell, 165, 1280–1292. 27203113 10.1016/j.cell.2016.04.038 PMC4907330

[pld319-bib-0052] Petukhova, G. , Van Komen, S. , Vergano, S. , Klein, H. , & Sung, P. (1999). Yeast Rad54 promotes Rad51‐dependent homologous DNA pairing via ATP hydrolysis‐driven change in DNA double helix conformation. Journal of Biological Chemistry, 274, 29453–29462. 10506208 10.1074/jbc.274.41.29453

[pld319-bib-0053] Prelich, G. (2012). Gene overexpression: Uses, mechanisms, and interpretation. Genetics, 190, 841–854. 22419077 10.1534/genetics.111.136911 PMC3296252

[pld319-bib-0054] Robbins, M. L. , Sekhon, R. S. , Meeley, R. , & Chopra, S. (2008). A Mutator transposon insertion is associated with ectopic expression of a tandemly repeated multicopy Myb gene pericarp color1 of maize. Genetics, 178, 1859–1874. 18430921 10.1534/genetics.107.082503 PMC2323782

[pld319-bib-0055] Sekhon, R. S. , Lin, H. , Childs, K. L. , Hansey, C. N. , Buell, C. R. , de Leon, N. , & Kaeppler, S. M. (2011). Genome‐wide atlas of transcription during maize development. Plant Journal, 66, 553–563. 21299659 10.1111/j.1365-313X.2011.04527.x

[pld319-bib-0056] Settles, A. M. , Holding, D. R. , Tan, B. C. , Latshaw, S. P. , Liu, J. , Suzuki, M. , … McCarty, D. R. (2007). Sequence‐indexed mutations in maize using the UniformMu transposon‐tagging population. BMC Genomics, 8, 116. 17490480 10.1186/1471-2164-8-116 PMC1878487

[pld319-bib-0057] Sidorenko, L. , Dorweiler, J. E. , Cigan, A. M. , Arteaga‐Vazquez, M. , Vyas, M. , Kermicle, J. , Jurcin, D. , Brzeski, J. , Cai, Y. , & Chandler, V. L. (2009). A dominant mutation in mediator of paramutation2, one of three second‐largest subunits of a plant‐specific RNA polymerase, disrupts multiple siRNA silencing processes. PLoS Genetics, 5, e1000725. 19936058 10.1371/journal.pgen.1000725 PMC2774164

[pld319-bib-0058] Sims, H. I. , Baughman, C. B. , & Schnitzler, G. R. (2008). Human SWI/SNF directs sequence‐specific chromatin changes on promoter polynucleosomes. Nucleic Acids Research, 36, 6118–6131. 18820294 10.1093/nar/gkn623 PMC2577362

[pld319-bib-0059] Sims, H. , Lane, J. M. , Ulyanova, N. P. , & Schnitzler, G. (2007). Human SWI/SNF drive sequence‐directed repositioning of nucleosomes on C‐MYC promoter DNA minicircles. Biochemistry, 46, 11377–11388. 17877373 10.1021/bi7008823 PMC2526049

[pld319-bib-0060] Sloan, A. E. , Sidorenko, L. , & McGinnis, K. M. (2014). Diverse gene‐silencing mechanisms with distinct requirements for RNA polymerase subunits in Zea mays. Genetics, 198, 1031–1042. 25164883 10.1534/genetics.114.168518 PMC4224150

[pld319-bib-0061] Smith, L. M. , Pontes, O. , Searle, I. , Yelina, N. , Yousafzai, F. K. , Herr, A. J. , … Baulcombe, D. C. (2007). An SNF2 protein associated with nuclear RNA silencing and the spread of a silencing signal between cells in *Arabidopsis* . Plant Cell, 19, 1507–1521. 17526749 10.1105/tpc.107.051540 PMC1913737

[pld319-bib-0062] Stam, M. , Belele, C. , Dorweiler, J. E. , & Chandler, V. L. (2002). Differential chromatin structure within a tandem array 100 kb upstream of the maize b1 locus is associated with paramutation. Genes & Development, 16, 1906–1918. 12154122 10.1101/gad.1006702 PMC186425

[pld319-bib-0063] Stam, M. , Belele, C. , Ramakrishna, W. , Dorweiler, J. E. , Bennetzen, J. L. , & Chandler, V. L. (2002). The regulatory regions required for B' paramutation and expression are located far upstream of the maize b1 transcribed sequences. Genetics, 162, 917–930. 12399399 10.1093/genetics/162.2.917 PMC1462281

[pld319-bib-0064] Stonaker, J. L. , Lim, J. P. , Erhard, K. F. , & Hollick, J. B. (2009). Diversity of Pol IV function is defined by mutations at the maize rmr7 locus. PLoS Genetics, 5, e1000706. 19936246 10.1371/journal.pgen.1000706 PMC2775721

[pld319-bib-0065] Tamura, K. , Stecher, G. , Peterson, D. , Filipski, A. , & Kumar, S. (2013). MEGA6: Molecular evolutionary genetics analysis version 6.0. Molecular Biology, 30(12), 2725–2729. 10.1093/molbev/mst197 PMC3840312 24132122

[pld319-bib-0066] Till, B. J. , Reynolds, S. H. , Weil, C. , Springer, N. , Burtner, C. , Young, K. , … Henikoff, S. (2004). Discovery of induced point mutations in maize genes by TILLING. BMC Plant Biology, 4, 12. 15282033 10.1186/1471-2229-4-12 PMC512284

[pld319-bib-0501] Vera, D. L. , Madzima, T. F. , Labonne, J. D. , Alam, M. P. , Hoffman, G. G. , Girimurugan, S. B. , … Bass, H. W. (2014). Differential nuclease sensitivity profiling of chromatin reveals biochemical footprints coupled to gene expression and functional DNA elements in maize. Plant Cell, 26(10), 3883–3893. 25361955 10.1105/tpc.114.130609 PMC4247582

[pld319-bib-0067] Wendte, J. M. , & Pikaard, C. S. (2016). The RNAs of RNA‐directed DNA methylation. Biochim Biophys Acta ‐ Gene Regulatory Mechanisms, 1860(1), 140–148. 27521981 10.1016/j.bbagrm.2016.08.004 PMC5203809

[pld319-bib-0068] Yu, C. , Chen, S. C.‐C. , Chang, Y. , Liu, W.‐Y. , Lin, H.‐H. , Lin, J.‐J. , … Li, W.‐H. (2015). Transcriptome dynamics of developing maize leaves and genomewide prediction of cis elements and their cognate transcription factors. PNAS, 112(19), E2477–E2486. 25918418 10.1073/pnas.1500605112 PMC4434728

[pld319-bib-0069] Zhang, H. , Ma, Z.‐Y. , Zeng, L. , Tanaka, K. , Zhang, C.‐J. , Ma, J. , … Zhu, J. K. (2013). DTF1 is a core component of RNA‐directed DNA methylation and may assist in the recruitment of Pol IV. PNAS, 110, 8290–8295. 23637343 10.1073/pnas.1300585110 PMC3657815

