# Supplementary material for: Altered nucleosome positions in maize haplotypes and mutants of a subset of SWI/SNF‐like proteins
Source: Plant Direct. 2017 Oct 16;1(4):e00019. doi: 10.1002/pld3.19 (PMC6508530; doi:10.1002/pld3.19)
Supplement: Supplementary file 1 [file PLD3-1-e00019-s001.pdf]

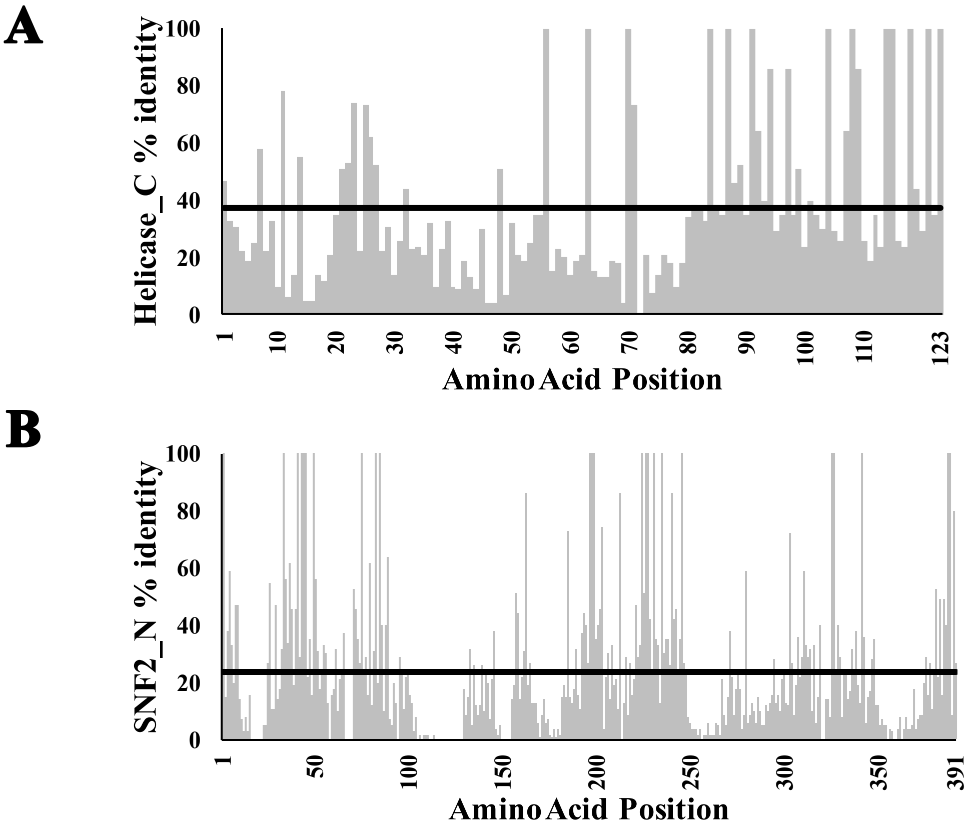

Figure S1. The conservation of Helicase_C and SNF2_N domains. A, The % identity of the Helicase_C domain across ScSNF2, ScRAD54, AtDDM1, AtDRD1, AtCLSY1, CHR101, CHR106, CHR127, CHR156, RMR1, CHR167, GRMZM2G102625, GRMZM2G083138, and GRMZM2G108166 was calculated at each MUSCLE aligned amino acid position (Amino Acid Position) and is indicated (gray bars). The overall average % identity across all amino acids was calculated (black line) at 37%. B, The % identity of the SNF2_N domain across ScSNF2, ScRAD54, AtDDM1, AtDRD1, AtCLSY1, CHR101, CHR106, CHR127, CHR156, RMR1, CHR167, GRMZM2G102625, GRMZM2G083138, and GRMZM2G108166 was calculated at each MUSCLE aligned amino acid position (Amino Acid Position) and is indicated (gray bars). The overall average % identity across all amino acids was calculated (black line) at 24%.

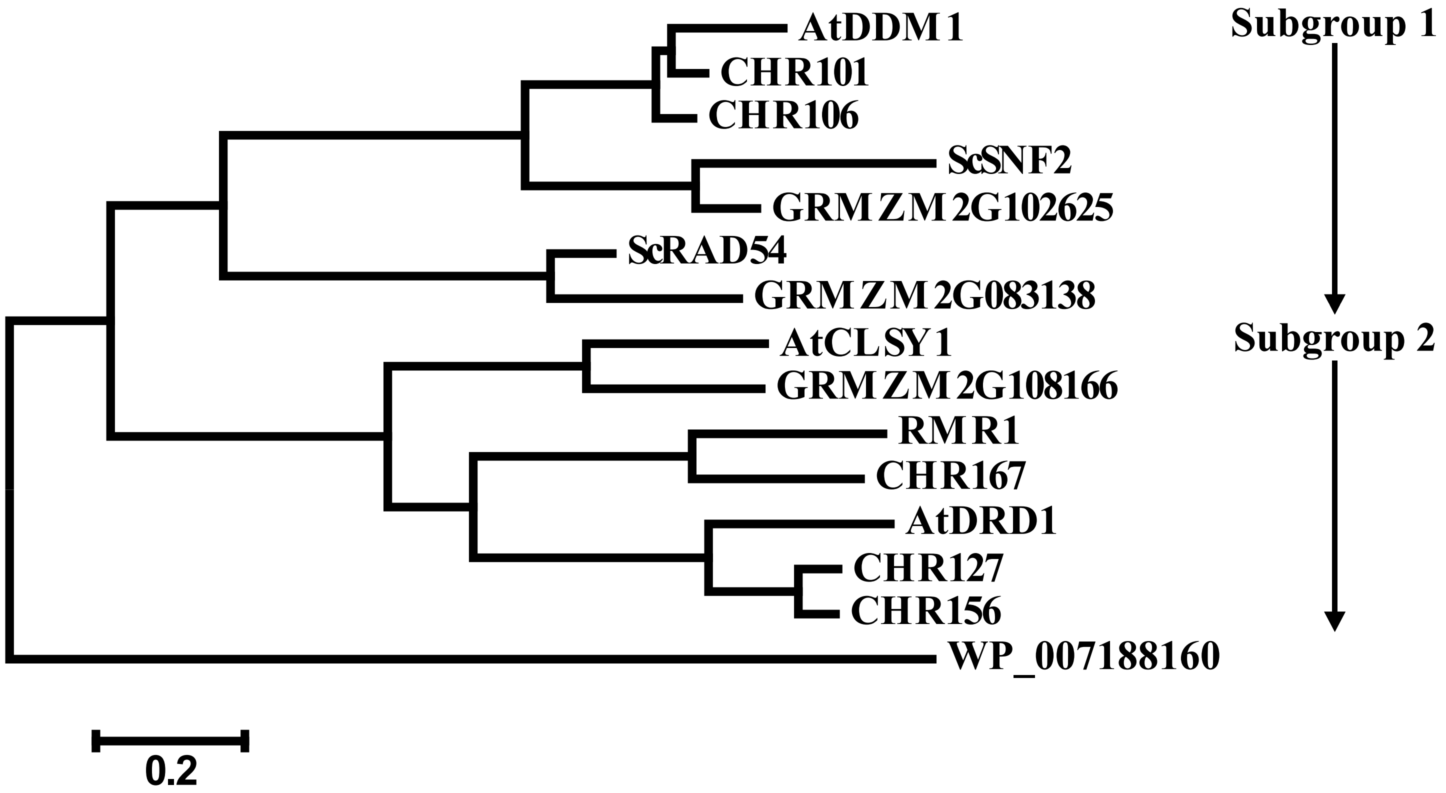

Figure S2. Phylogenetic relationship of the Helicase_C domain of maize, *A. thaliana*, and *S. cerevisiae* chromatin proteins. A phylogenetic tree of the maize orthologs of the *S. cerevisiae* (Sc) and *A. thaliana* (At) chromatin proteins was constructed using MEGA7's Maximum Likelihood method based on the JTT matrix-based model. The Helicase_C domain of Archaea *Haloarcula californiae* ATP-dependent Helicase (WP_007188160) was used as the outgroup. A branch length scale bar is indicated, in number of amino acid substitutions per site. Two subgroups were identified.

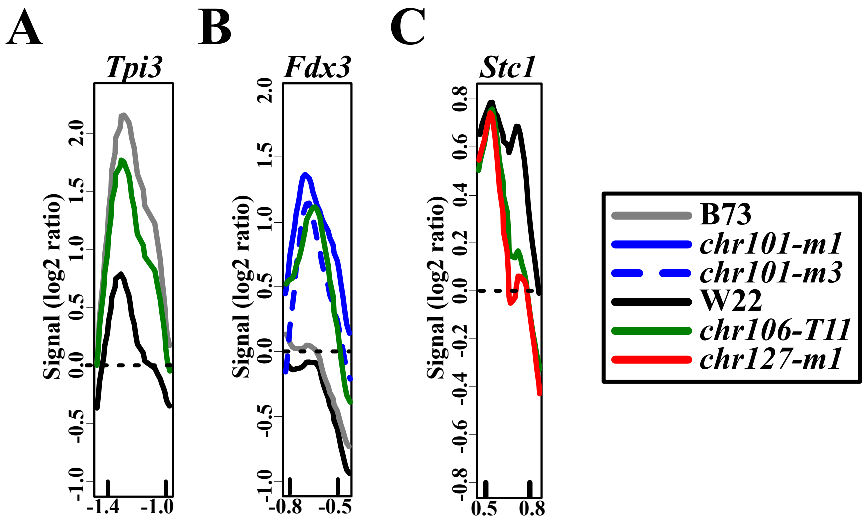

Figure S3. Nucleosome position at loci with differences in multiple genotypes. Genes are all oriented according to a left to right transcriptional direction, with the nucleotide position in kb relative to the transcription start site (0) indicated on the x-axes. Relative fluorescent signals are indicated on the y-axes (Signal (log2 ratio)). A, Comparison of nucleosome position at *Tpi3_NP1* between wild type B73, wild type W22, and homozygous *chr106-T11* plants. B, Comparison of nucleosome position at *Fdx3_NP1* between wild type B73, wild type W22, homozygous *chr101-m1*, homozygous *chr101-m3*, and homozygous *chr106-T11* plants. C, Comparison of nucleosome position at *Stc1_NP1* between wild type W22, homozygous *chr106-T11*, and homozygous *chr127-m1* plants. Three pools of three biological replicates were averaged for each plot, except for *chr101-m3*, which includes one pool of three biological replicates.

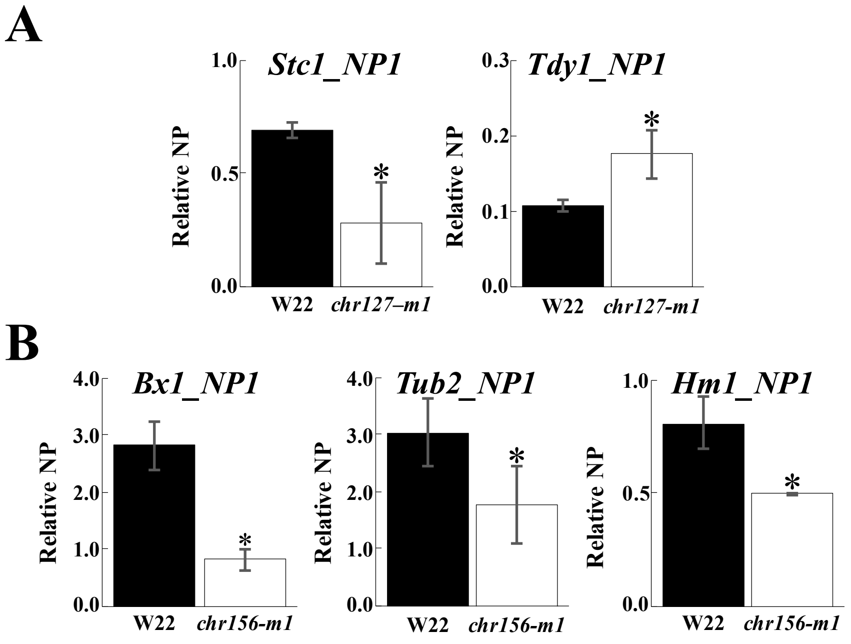

Figure S4. MNase-qPCR analysis of nucleosome position (NP) in homozygous *chr127-m1* and *chr156-m1* plants. Relative $2^{-\Delta\Delta Ct}$ values (Relative NP) are indicated on the y-axes. A, Wild type W22 (black bar) and homozygous *chr127-m1* (white bar) plants are compared at *Stc1_NP1* and *Tdy1_NP1*. The data represents averages ±SD (error bars) of three pools of three biological replicates. Student t-test (p≤0.05) was used to identify statistically significant changes between mutant and WT (*). B, Wild type W22 (black bar) and homozygous *chr156-m1* (white bar) plants are compared at *Bx1_NP1*, *Tub2_NP1*, and *Hm1_NP1*. The data represents averages ±SD (error bars) of three pools of three biological replicates. Student t-test (p≤0.05) was used to identify statistically significant changes between mutant and WT (*).

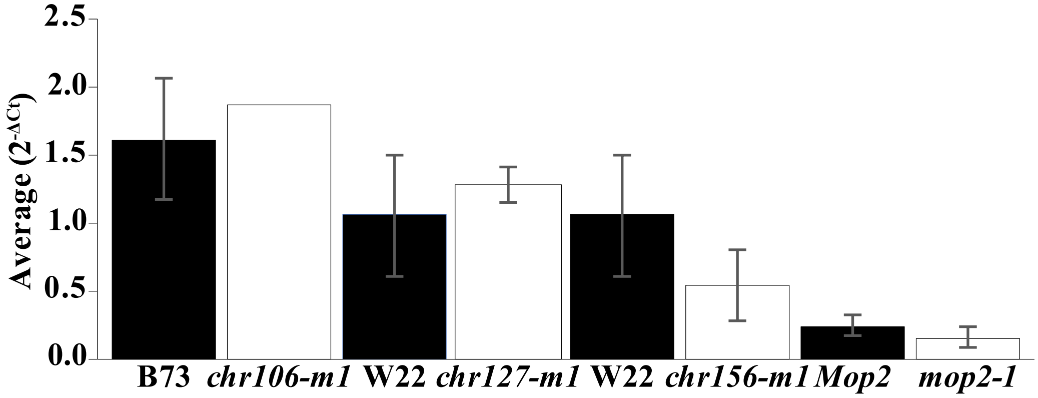

Figure S5. MNase-qPCR analysis of nucleosome position at *Mwp1*. Comparison of $2^{-\Delta Ct}$ values for wild type B73 (black bar), homozygous *chr106-m1* (white bar), wild type W22 (black bar), homozygous *chr127-m1* (white bar), wild type W22 (black bar), homozygous *chr156-m1* (white bar), wild type *Mop2* (black bar), and homozygous *mop2-1* (whilte bar) plants at the control locus, *Mwp1*. The data represents averages ±SD (error bars) of three pools of three biological replicates each for all genotypes, except for *chr106m1*, which includes one pool of three biological replicates. No statistically significant changes between mutant and wild type were identified by Student t-test ($p \leq 0.05$).
