# Supplementary material for: Altered nucleosome positions in maize haplotypes and mutants of a subset of SWI/SNF‐like proteins
Source: Plant Direct. 2017 Oct 16;1(4):e00019. doi: 10.1002/pld3.19 (PMC6508530; doi:10.1002/pld3.19)
Supplement: Supplementary file 2 [file PLD3-1-e00019-s002.pdf]

Table S1. Maize genes with % identity (ID) to *S. cerevisiae* (Sc) or *A. thaliana* (At) chromatin remodelers.

Gene Name	Gene ID	ScSNF2 % Identity	ScRAD54 % Identity	AtDDM1 % Identity	AtDRD1 % Identity	AtCLSY1 % Identity
CHR101	GRMZM2G177165	**43**	30	**65**	24	24
CHR106	GRMZM2G071025	**43**	29	**65**	26	**33**
CHR127	GRMZM5G574858	26	23	24	**54**	29
CHR156	GRMZM2G393742	25	23	24	**54**	29
RMR1	GRMZM2G154946	23	24	24	31	**33**
CHR167	GRMZM2G178435	24	24	28	30	**32**
Unnamed	GRMZM2G083138	30	**42**	29	26	25
	GRMZM2G102625	**47**	33	45	30	28
	GRMZM2G108166	26	24	23	30	**36**

Maize protein within the top 5% identities (Altschul et al., 1990) to ScSNF2, ScRAD54, AtDDM1, AtDRD1, and AtCLSY1 are in bold.

Table S2. The alleles and their genetic backgrounds, the primer IDs, names, and sequences along with the detection methods used for the genotyping of the maize mutants are listed

Allele	Genetic background	Primer ID	Primer name	Primer sequence (5' to 3')	Detection method
chr101-m1	B73	KM1210	chr101_F1	AAAGCTTCCGTTTCCTTCAGTTCAC	PCR with F/R for wild type and F/TIR6 for transposon insertion.
		KM1211	chr101_R1	CTCCAGTAGTCGCTCTTCCTCCTTC	
chr101-m3	B73	KM1212	chr101_F2	GAAGAGGCTGCTAGACTTGCTTTTG	PCR with F/R for wild type and F/TIR6 for transposon insertion.
		KM1213	chr101_R2	TCTTCTACCTGTGGCTGTTCAGCTTGAG	
chr106-T11	W22	KM89	chr106_F1	ATTGCAGGGATGATGATGTGCTGGTT	PCR with F/R then MwoI (NEB) digest. Mutants missing one of the bands.
		KM90	chr106_R1	TGCACCCAGTCATTGGAATTTGTGTC	
chr106-m1	B73	KM1075	chr106_F2	GACGAAGACCTCTTGAAGCTGATG	PCR with F/R for wild type and F/TIR6 for transposon insertion.
		KM1076	chr106_R2	TACATCATAGAACGCAGCAACAGAAG	
chr127-m1	W22	KM1206	chr127_F1	TCGAGCACCCACCCACCTCACTC	PCR with F/R for wild type and F/TIR6 for transposon insertion.
		KM1207	chr127_R1	GCAACAGGGAGTGCATGACCACTG	
chr156-m1	W22	KM1208	chr156_F1	GTCCCGGGCCCTTCTCCTTCCT	PCR with F/R for wild type and F/TIR6 for transposon insertion.
		KM1209	chr156_R1	TCCCGTCCTCCCGGTGACTGAG	
rmr1-1	Mixed	KM1120	rmr1-1_F1	GTGTCAGTGTTTGCCGAGAA	PCR with F/R, then sequence with nested F primer.
		KM1203	rmr1-1_R1	TGACTCAGTTGGTGGATAATGG	
		KM1202	rmr1-1_nested_F2	AGGTGGAAAACATTGGTTCTG	
mop2-1	W23/K55	KM550	mop2-1_F1	ATGTCCGAAGATAAGGTGAAAT	PCR with F/R, then sequence with F primer.
		KM551	mop2-1_R1	AGATCCCCATGCTGAAGAGT	
TIR6		KM476	MuTIR 6	AGAGAAGCCAACGCCAWCGCCTCYATTTCGTC	

Table S3. The primer targets, IDs, names, and sequences used for the qRT-PCR.

Primer Target	Primer ID	Primer name	Primer sequence (5' to 3')	Amplicon (bp)
chr101-m1	KM1298	chr101_F3	TCAACCTTACTGCCGCTGACAC	157
	KM1589	chr101_R3	ATCATCCGCCCCTCAACG	
chr101-m3	KM1298	chr101_F3	TCAACCTTACTGCCGCTGACAC	69
	KM1299	chr101_R4	CCTGCAAATCCATCTGAGGGTTCC	
chr106-T11	KM1325	chr106_F3	GCTCACCAGCTGAGCGATGC	112
	KM1326	chr106_R3	AAAATATCCGCCTGATCGGC	
chr106-m1	KM1325	chr106_F3	GCTCACCAGCTGAGCGATGC	112
	KM1326	chr106_R3	AAAATATCCGCCTGATCGGC	
chr127-m1	KM1592	chr127_F2	GTTCTTGATTCAGACGATGAGG	93
	KM1582	chr127_R2	AATCAATCCAGATGTTCCGAG	
chr156-m1	KM1498	chr156_F2	CCATCCAACACTGGGGGAGT	121
	KM1593	chr156_R2	GAGACATCGGTGAGTTGATTGC	
Stc1	KM1444	Stc1_F2	CCGAGTGTATAGAAAGCTGGGACC	113
	KM1445	Stc1_R2	CGCTAACTCTTCCTCAATGCTTTG	
Tdy1	KM1420	Tdy1_F1	GTCGTCCCGTCAGTGTCCAT	157
	KM1422	Tdy1_R1	AAACCCCCGTTTCGTAGTCA	
Bx1	KM1448	Bx1_F2	GAGCACGTGAAGCAGATTGCG	125
	KM1449	Bx1_R2	CTGGCATACTCCTCCAGCCT	
Hm1	KM1452	Hm1_F2	GCGTACCCGACGATCCACG	122
	KM1453	Hm1_R2	AGCTCGCCCAACCTGTCC	
Ubiquitin conjugating enzyme	KM633	Ubi_F1	GACTACACGATGGAGAACATCCTAACCC	96
	KM634	Ubi_R1	GAAGAATGTCCCTTCTGGAGGCTGC	
45S	KM1406	45S_F1	TTGAGAAGTGCTTGCGTGC	322
	KM1407	45S_R1	GTAGCACGTCCTCGCAGAC	

Table S4. The primer targets IDs, names, and sequences used for MNase-qPCR.

Primer Target	Primer ID	Primer name	Primer sequence (5' to 3')	Amplicon (bp)
Stc1	KM1416	Stc1_F1	GCTGTAGACCATGGGCTAGGAG	113
	KM1418	Stc1_R1	CGAGGGTGTCTGGCGATCT	
Tdy1	KM1420	Tdy1_F1	GTCGTCCCGTCAGTGTCCAT	157
	KM1422	Tdy1_R1	AAACCCCCGTTTCGTAGTCA	
Hm1	KM1433	Hm1_F1	ACCACAGGCCGACAACCG	100
	KM1434	Hm1_R1	TCTGGCATGAGAAAGCTGGG	
Bx1	KM1424	Bx1_F1	GAGCCGAGCATCGATCCAG	92
	KM1427	Bx1_R1	CCATGTGAGTGAATCTCACCTCA	
Tub2	KM1429	Tub2_F1	TATAGGGTTCGACCACCGTGT	112
	KM1430	Tub2_R1	ATGGGTACCCTGCTCATTTCT	
b1tr-R3	KM1602	B1TR_F1	CAAGATCCATTGAACATCTTGTCC	116
	KM1603	B1TR_R1	CATGTGTGAGGGTGATGCTGCG	
Fdx3	KM1596	Fdx3_F1	TGAGGCTGGAAACCTGGG	69
	KM1597	Fdx3_R1	CCCTTTCGGTCAGAGGGAG	
Mwp1	KM1436	Mwp1_F1	TGGAGCAGTGCACCCTTTTT	128
	KM1438	Mwp1_R1	TCCAATCTGAAAGCAGTACTGGG	
