# Supplementary material for: Altered nucleosome positions in maize haplotypes and mutants of a subset of SWI/SNF‐like proteins
Source: Plant Direct. 2017 Oct 16;1(4):e00019. doi: 10.1002/pld3.19 (PMC6508530; doi:10.1002/pld3.19)
Supplement: Supplementary file 3 [file PLD3-1-e00019-s003.pdf]

Reviewer Comments:
Reviewer #1:
None

Reviewer #2:
Comments to the Author (Required):
The authors have largely addressed my concerns and suggestions and I now recommend this manuscript for publication in Plant Direct.
On a side note: The decision letter explicitly asked for a rebuttal letter that explains how the points were addressed. Such a letter was unfortunately not provided. It helps the reviewer and editor assess the work even if the revisions requested were relatively minor.
